# Hematopoietic stem cell–derived Tregs are essential for maintaining favorable B cell lymphopoiesis following posttransplant cyclophosphamide

**DOI:** 10.1172/jci.insight.162180

**Published:** 2023-04-24

**Authors:** Yuichi Sumii, Takumi Kondo, Shuntaro Ikegawa, Takuya Fukumi, Miki Iwamoto, Midori Filiz Nishimura, Hiroyuki Sugiura, Yasuhisa Sando, Makoto Nakamura, Yusuke Meguri, Takashi Matsushita, Naoki Tanimine, Maiko Kimura, Noboru Asada, Daisuke Ennishi, Yoshinobu Maeda, Ken-ichi Matsuoka

**Affiliations:** 1 Department of Hematology, Oncology and Respiratory Medicine and; 2 Department of Pathology, Okayama University Graduate School of Medicine, Dentistry and Pharmaceutical Sciences, Okayama, Japan.; 3 Department of Dermatology, Faculty of Medicine, College of Medical, Pharmaceutical and Health Sciences, Kanazawa University, Kanazawa, Japan.; 4 Department of Gastroenterological and Transplant Surgery, Graduate School of Biomedical and Health Sciences, Hiroshima University, Hiroshima, Japan.; 5 Department of Hematology and Oncology, Okayama University Hospital, Okayama, Japan.

**Keywords:** Hematology, Transplantation, Bone marrow transplantation, Mouse models, Tolerance

## Abstract

Posttransplant cyclophosphamide (PTCy) is associated with a low incidence of chronic graft-versus-host disease (cGVHD) following hematopoietic stem cell (HSC) transplantation. Previous studies have shown the important roles of B cell immunity in cGVHD development. Here, we investigated the long-term reconstitution of B lymphopoiesis after PTCy using murine models. We first demonstrated that the immune homeostatic abnormality leading to cGVHD is characterized by an initial increase in effector T cells in the bone marrow and subsequent B and Treg cytopenia. PTCy, but not cyclosporine A or rapamycin, inhibits the initial alloreactive T cell response, which restores intra-bone marrow B lymphogenesis with a concomitant vigorous increase in Tregs. This leads to profound changes in posttransplant B cell homeostasis, including decreased B cell activating factors, increased transitional and regulatory B cells, and decreased germinal center B cells. To identify the cells responsible for PTCy-induced B cell tolerance, we selectively depleted Treg populations that were graft or HSC derived using DEREG mice. Deletion of either Treg population without PTCy resulted in critical B cytopenia. PTCy rescued B lymphopoiesis from graft-derived Treg deletion. In contrast, the negative effect of HSC-derived Treg deletion could not be overcome by PTCy, indicating that HSC-derived Tregs are essential for maintaining favorable B lymphopoiesis following PTCy. These findings define the mechanisms by which PTCy restores homeostasis of the B cell lineage and reestablishes immune tolerance.

## Graphical abstract

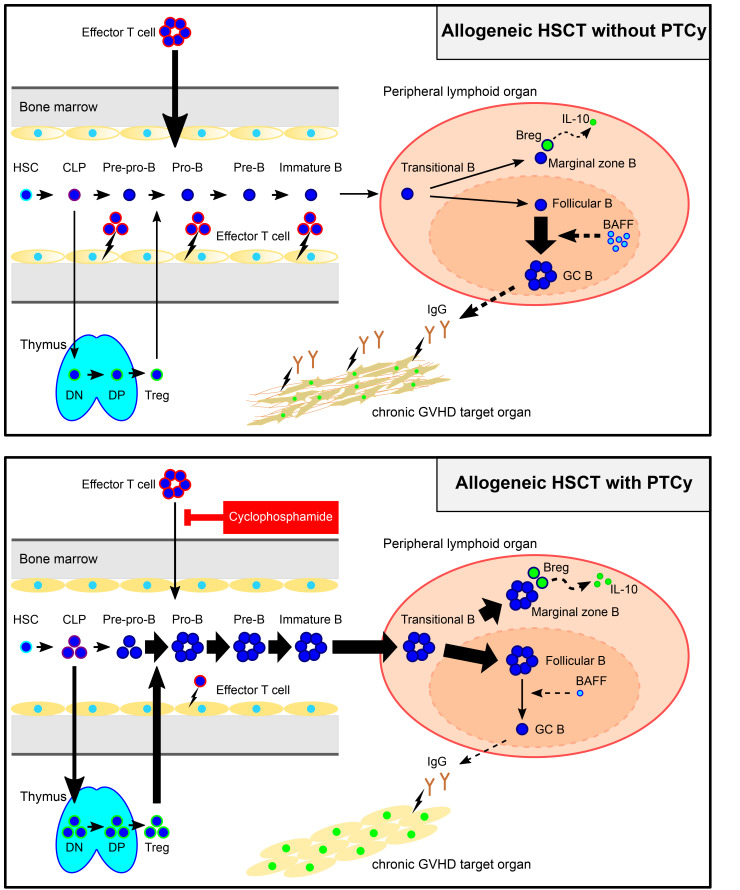

## Introduction

Allogeneic hematopoietic stem cell transplantation (HSCT) is a curative treatment for otherwise-incurable hematological diseases, including malignancies and bone marrow failure syndromes. However, chronic graft-versus-host disease (GVHD), which has clinical manifestations resembling those of autoimmune diseases (1, 2), is a major complication of allogeneic HSCT that can cause morbidity and nonrelapse mortality in long-term survivors (3–5).

CD4^+^CD25^+^Foxp3^+^ Tregs play an indispensable role in maintaining tolerance after allogeneic HSCT (6, 7). Patients with chronic GVHD have a reduced frequency of Tregs (6), and long-term impaired reconstitution of Tregs is associated with the incidence of extensive chronic GVHD (7). IL-2 is critical for Treg homeostasis (8, 9), and administration of a low dose of IL-2 to patients with active chronic GVHD restores Treg homeostasis (10) and ameliorates clinical symptoms (11). A preclinical study demonstrated that the specific depletion of donor Tregs induces cutaneous chronic GVHD and that adoptive transfer of donor Tregs attenuates skin pathology (12).

Tregs are also important for B cell differentiation from hematopoietic stem cells (HSCs) by maintaining immunological homeostasis in the bone marrow microenvironment (13). For allogeneic bone marrow transplantation (BMT), the administration of splenic donor Tregs improved B cell development in the bone marrow (14).

Posttransplant B cell immunity, coordinated with donor T cells and pathological antibodies, is also associated with the development of chronic GVHD (15–19). B cell homeostasis is altered in patients with chronic GVHD (20) and is associated with high B cell activating factor (BAFF)/B cell ratios (21, 22). Peripheral B cells from patients with chronic GVHD show increased proliferation in response to B cell receptor (BCR) stimulation along with elevated proximal BCR signaling (23). Intriguingly, the pathological production of BAFF after allogeneic BMT increases the frequency of BCR-activated B cells and anti-recipient IgG production (24). For clinical application, Dubovsky et al. demonstrated that ibrutinib, a Bruton’s tyrosine kinase inhibitor, ameliorated experimental chronic GVHD, indicating that B cell homeostatic abnormalities could be a therapeutic target for chronic GVHD (25). Shortly thereafter, ibrutinib was tested in a clinical trial and was approved by the FDA for the treatment of chronic GVHD (26).

Although persistent excess BAFF after transplantation, which is involved in altered B cell homeostasis, is potentially a consequence of delayed B cell reconstitution in the peripheral blood (20, 27), intra–bone marrow B cell development in the context of chronic GVHD has not yet been studied in detail. A decreased number of B cell precursors in the bone marrow after allogeneic HSCT is associated with the incidence of acute and chronic GVHD (28, 29). Furthermore, T cell infiltration into the bone marrow within the first month is associated with reduced numbers of osteoblasts in the same period and delayed B cell recovery in the peripheral blood within 6 months after allogeneic HSCT (30). In murine models, the loss of osteoblasts and suppression of B lymphopoiesis in the bone marrow were observed during the first month after BMT (14, 31).

In the past decade, HLA-haploidentical HSCT with posttransplant cyclophosphamide (PTCy) has been developed for patients who lack HLA-matched donors and/or require urgent transplantation. This strategy uses T cell–replete grafts followed by cyclophosphamide (32–35). Clinical studies have demonstrated that the survival outcomes are comparable to those of HLA-matched transplantation with conventional acute GVHD prophylaxis (34, 35). Notably, these studies revealed that PTCy-based HLA-haploidentical HSCT is associated with a significantly reduced risk of chronic GVHD as well as severe acute GVHD (34, 35).

The mechanism by which PTCy prevents acute GVHD involves the dysfunction of alloreactive proliferative effector T cells (36) while preserving donor Tregs (36–38). In the context of chronic GVHD, previous clinical studies have reported rapid B cell recovery in the peripheral blood following PTCy-based GVHD prophylaxis compared with conventional GVHD prophylaxis (39, 40) and that PTCy restores favorable B cell homeostasis with a predominant naive B phenotype (39, 41). Crucially, we demonstrated that early recovery of the naive B cell pool is significantly associated with a low incidence of subsequent chronic GVHD (39).

These findings suggest a possible relationship between early T cell immunity and subsequent B cell reconstitution. However, the detailed mechanism by which early T cell immunity alters B cell immunity and induces chronic GVHD remains unclear. To address this issue, we assessed longitudinal B cell reconstitution, including intra–bone marrow B cell development and peripheral B cell maintenance, using murine BMT models to clarify the mechanisms responsible for the pathogenesis of clinical chronic GVHD and the impact of early T cell homeostasis by PTCy on subsequent immune reconstitution. Although several murine models of chronic GVHD have been reported (42, 43), here we adopted the C57BL/6-into-B6D2F1 (BDF1) system, which is commonly used as a model of acute GVHD, to continuously monitor the impact of acute T cell inflammation on immune reconstitution from the early to late phase of transplantation. A previous study demonstrated that BDF1 recipients transplanted with C57BL/6 grafts, even without granulocyte-colony stimulating factor treatment, developed multiorgan tissue fibrosis as a pathological feature of chronic GVHD after long-term survival when the total T cell concentration in the graft was reduced (12). Recently, we verified that this system could be used as a model for PTCy-based MHC-haploidentical BMT (44).

## Results

### Graft-derived effector T cells increased in the bone marrow, and B lymphopoiesis was suppressed during the first month after allogeneic BMT.

**Figure 1:**
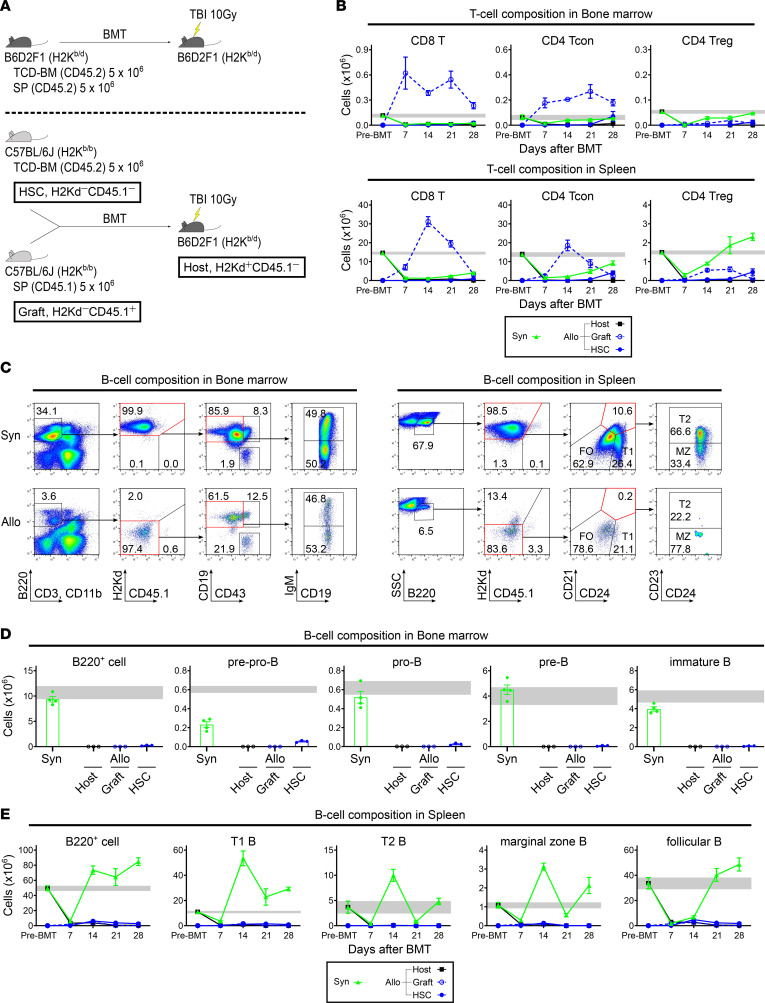
Graft-derived effector T cells increase in the bone marrow and B lymphopoiesis is suppressed during the first month after allogeneic BMT. (**A**) Lethally irradiated (10 Gy) BDF1 recipients (H2K^b/d^CD45.2^+^) received transplants of 5 × 10^6^ Ly 5.1 B6 (H2K^b/b^CD45.1^+^) splenocytes with 5 × 10^6^ B6 (H2K^b/b^CD45.2^+^) TCD-BM cells (allogeneic group, *n* = 12). The syngeneic group was administered the same numbers of splenocytes and TCD-BM cells from BDF1 mice (*n* = 15). Animals were euthanized on days 7, 14, 21, and 28 after allogeneic BMT to harvest bone marrow and spleens. T and B cell subsets in the allogeneic group were separated into host- (H2Kd^+^CD45.1^−^), graft- (H2Kd^−^CD45.1^+^), and HSC- (H2Kd^−^CD45.1^−^) derived cells using flow cytometry, respectively. (**B**) Kinetics of CD8^+^ T cell, CD4^+^ Tcon, and CD4^+^ Treg recovery in the bone marrow and spleen after BMT. (**C**) Representative flow cytometry plots identifying B220^+^ cell subsets and chimerism in the bone marrow and spleen of syngeneic and allogeneic groups on day 28 after BMT. (**D**) Numbers of B220^+^, pre-pro-B (B220^+^CD43^+^CD19^−^IgM^−^), pro-B (B220^+^CD43^+^CD19^+^IgM^−^), pre-B (B220^+^CD43^−^CD19^+^IgM^−^), and immature B (B220^+^CD43^−^CD19^+^IgM^+^) cells in the bone marrow on day 28 after BMT. (**E**) Kinetics of B220^+^, T1 B (B220^+^CD21^lo^CD24^hi^), T2 B (B220^+^CD21^hi^CD23^+^CD24^int^), marginal zone B (B220^+^CD21^hi^CD23^−^CD24^int^), and follicular B (B220^+^CD21^lo^CD24^lo^) cells’ recovery in the spleen after BMT. Gray bars (**B**, **D**, and **E**) indicate the mean reference values ± SEM of normal controls (NC, *n* = 3). Data were obtained from 1 experiment and expressed as the mean ± SEM. BDF1, B6D2F1; B6, C57BL/6J; Ly5.1 B6, CD45.1 C57BL/6J; TCD-BM, T cell–depleted bone marrow; Tcon, conventional T cell; HSC, hematopoietic stem cell; T1, transitional 1; T2, transitional 2; MZ, marginal zone; FO, follicular; BMT, bone marrow transplantation; SP, splenocyte; TBI, total body irradiation; Syn, syngeneic; Allo, allogeneic.

First, we evaluated the reconstitution of T and B cells during the first month after allogeneic BMT. To distinguish between lymphocytes derived from recipients, donor splenocytes, and donor bone marrow cells in the bone marrow and spleen of recipients, we used the congenic markers H2Kd and CD45.1, as shown in Figure 1A and Supplemental Figure 1B; supplemental material available online with this article; https://doi.org/10.1172/jci.insight.162180DS1 Specifically, we used the MHC-haploidentical BMT model, in which lethally irradiated BDF1 mice (H2K^b/d^CD45.2^+^) were transplanted with splenocytes from CD45.1 C57BL/6J (Ly 5.1 B6, H2K^b/b^CD45.1^+^) mice and T cell–depleted bone marrow (TCD-BM) cells from C57BL/6J (B6, H2K^b/b^CD45.2^+^) mice on day 0 (Figure 1A). We determined H2Kd^+^CD45.1^−^, H2Kd^−^CD45.1^+^, and H2Kd^−^CD45.1^−^ gated cells using flow cytometry in the allogeneic recipients as “Host-,” “Graft-,” and “HSC-” derived cells, respectively (Figure 1A and Supplemental Figure 1). Syngeneic transplantation was performed as a control. Flow cytometric analysis revealed that graft-derived T cells increased not only in the spleen but also in the bone marrow of the allogeneic group following BMT (Supplemental Figure 1). Interestingly, the number of graft-derived effector T cells in the bone marrow was approximately 5 times the normal range and remained elevated during the first month after allogeneic BMT. However, the number of graft-derived Tregs did not increase (Figure 1B). One month after allogeneic BMT, HSC-derived T cells were detectable but remained much lower than those in the bone marrow of normal mice (Figure 1B and Supplemental Figure 1). B lymphopoiesis was evaluated in the bone marrow and spleen during the same period. In contrast to graft-derived T cells, the number of graft-derived B cells decreased immediately following BMT, and the number of HSC-derived pre-pro-B, pro-B, pre-B, immature B, T1 B, T2 B, marginal zone B, and follicular B cells in the allogeneic group barely increased until the end of the first month following BMT (Figure 1, C–E). In contrast, in the syngeneic group, Treg and B cell subsets, which decreased immediately after BMT, were restored to almost normal levels by the end of the first month after BMT (Figure 1, B–E, and Supplemental Figure 1).

### PTCy was associated with a decrease in graft-derived effector T cells in the bone marrow soon after BMT and with an increase in HSC-derived mature T cells in the later period.

**Figure 2:**
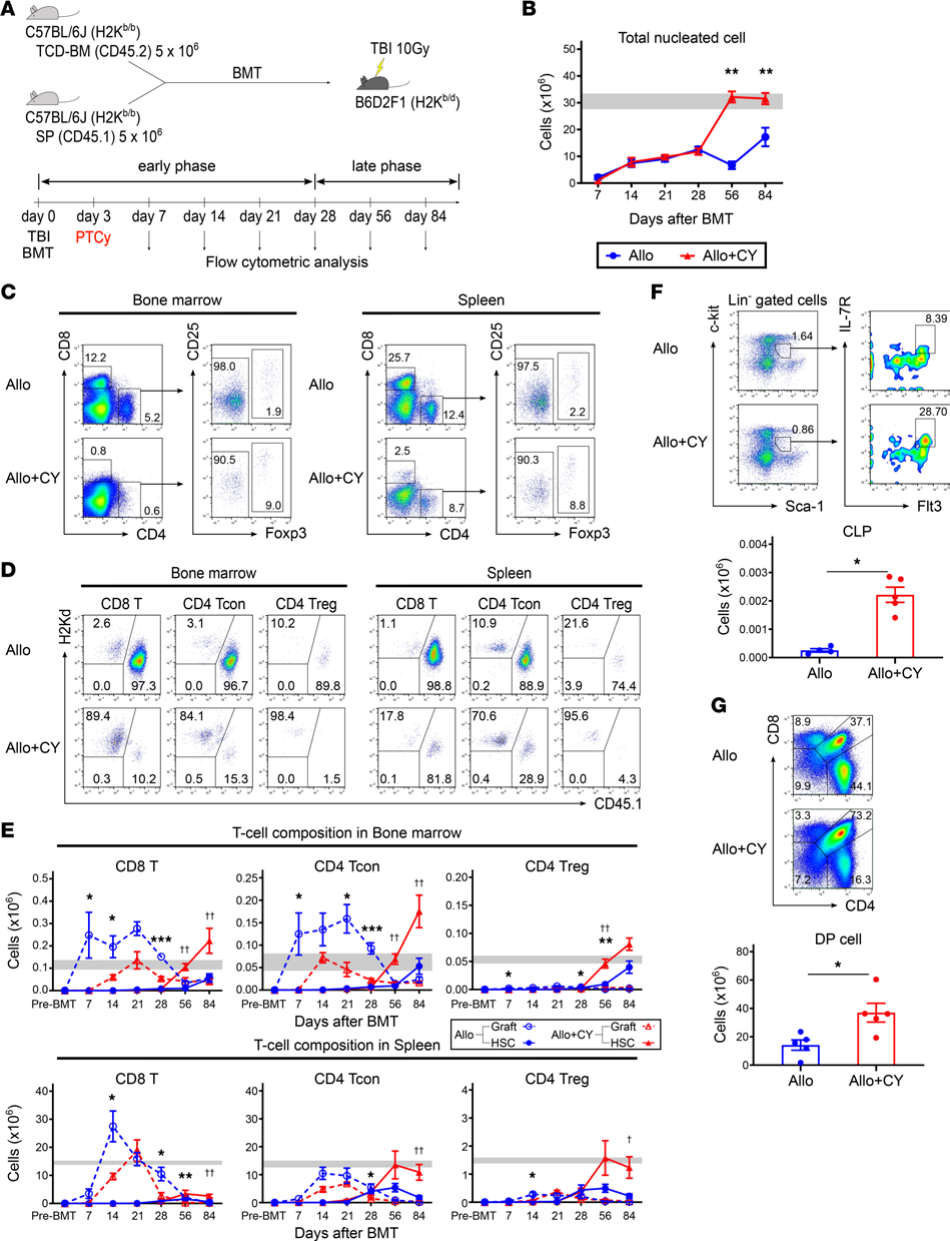
PTCy is associated with a decrease in graft-derived effector T cells in the bone marrow soon after BMT and increase in HSC-derived mature T cells in the later period. (**A**) Lethally irradiated (10 Gy) BDF1 recipients (H2K^b/d^CD45.2^+^) received transplants of 5 × 10^6^ Ly 5.1 B6 (H2K^b/b^CD45.1^+^) splenocytes with 5 × 10^6^ B6 (H2K^b/b^CD45.2^+^) TCD-BM cells. All recipient mice were injected intraperitoneally with 50 mg/kg of cyclophosphamide or vehicle on day 3 after allogeneic BMT (vehicle-treated, *n* = 30; and PTCy-treated, *n* = 32). Animals were euthanized on days 7, 14, 21, and 28 (early phase) and days 56 and 84 (late phase) after allogeneic BMT to harvest bone marrow, spleens, and thymi. (**B**) The kinetics of total nucleated cell recovery in the bone marrow after allogeneic BMT. (**C** and **D**) Representative flow cytometry plots identifying CD4^+^ and CD8^+^ T cell subsets (**C**) and chimerism (**D**) in the bone marrow and spleen on day 7 after allogeneic BMT. (**E**) Kinetics of graft- and HSC-derived CD8^+^ T cell, CD4^+^ Tcon, and CD4^+^ Treg recovery in the bone marrow and spleen after allogeneic BMT. * and ^†^ (**E**) indicate the comparison between graft-derived T cells in vehicle-treated group versus those in PTCy-treated group and HSC-derived T cells in vehicle-treated group versus those in PTCy-treated group, respectively. Gray bars (**B** and **E**) indicate mean reference values ± SEM of NC (*n* = 3). (**F**) Representative flow cytometry plots identifying CLP (Lin^−^c-Kit^int^Sca-1^int^IL-7Rα^+^Flt3^+^) cells and the total number of CLP cells in the bone marrow on day 56 after allogeneic BMT (vehicle-treated, *n* = 4, and PTCy-treated, *n* = 5). (**G**) Representative flow cytometry plots identifying DP (CD4^+^CD8^+^) cells and number of DP cells in the thymus on day 56 after allogeneic BMT (vehicle-treated, *n* = 5, and PTCy-treated, *n* = 5). Graft- and HSC-derived cells were defined as H2Kd^−^CD45.1^+^ and H2Kd^−^CD45.1^−^ gated cells by flow cytometry, respectively. Data from 2 independent experiments were combined and expressed as the mean ± SEM. *P* values were determined using the Mann-Whitney *U* test. *,^†^*P* < 0.05, **,^††^*P* < 0.01, ***P < 0.001. CY, cyclophosphamide; CLP, common lymphoid progenitor; DP, double-positive; Lin, lineage.

The effect of PTCy on the restoration of hematopoiesis was then investigated, both in the early (within 1 month) and late phases (1 month onward) following allogeneic BMT. As an experimental transplantation model that included PTCy treatment, lethally irradiated BDF1 mice were transplanted with splenocytes from Ly 5.1 B6 mice and TCD-BM cells from B6 mice on day 0, and cyclophosphamide was administered on day 3 (Figure 2A). We examined the complete blood cell count (CBC) in the peripheral blood and cellularity in the bone marrow every week during the first month and every month thereafter. The recovery of white blood cells, red blood cells, hemoglobin, and platelets in the peripheral blood was suppressed in the late phase after transplantation in the vehicle-treated group compared with the PTCy-treated group (Supplemental Figure 2). The difference between the 2 groups in the late phase was more evident in the recovery of bone marrow cellularity than in CBC (Figure 2B). We then examined the effects of PTCy on an increase in graft-derived T cells in the bone marrow and on T cell reconstitution after allogeneic BMT. After PTCy treatment, the number of graft-derived CD8^+^ T cells and CD4^+^ Tcons in the bone marrow and spleen was significantly lower than that in the vehicle-treated control group during the early phase after BMT (Figure 2, C–E, and Supplemental Figure 3). Interestingly, the effects of PTCy on graft-derived effector T cells were more evident in the bone marrow than in the spleen (Figure 2E and Supplemental Figure 3). Even in the late phase, the graft-derived effector T cells persisted longer in the vehicle-treated group than in the PTCy-treated group (Supplemental Figure 4). After the reduction in graft-derived T cells, HSC-derived T cells in the PTCy-treated group increased, and their numbers in the late phase after BMT were significantly higher than those in the vehicle-treated group (Figure 2E). With regard to Tregs, although the number of cells from donor grafts remained low, particularly in the bone marrow, during the early phase after BMT, HSC-derived Tregs increased significantly thereafter compared with the vehicle-treated group (Figure 2E). To further evaluate the development of lymphocytes from donor HSCs after BMT, we examined the development of T cell progenitors in the bone marrow and thymus on day 56 after allogeneic BMT. Flow cytometry analysis demonstrated that the number of common lymphoid progenitor (CLP) cells that differentiated into T and B cell lineages was significantly higher in the PTCy-treated group than in the vehicle-treated group (Figure 2F). In addition, PTCy promoted the recovery of CD4^+^CD8^+^ double-positive cells in the thymus of the recipient mice (Figure 2G).

### PTCy was associated with an increase in B cell progenitors in the bone marrow and mature B cells in the spleen after BMT.

**Figure 3:**
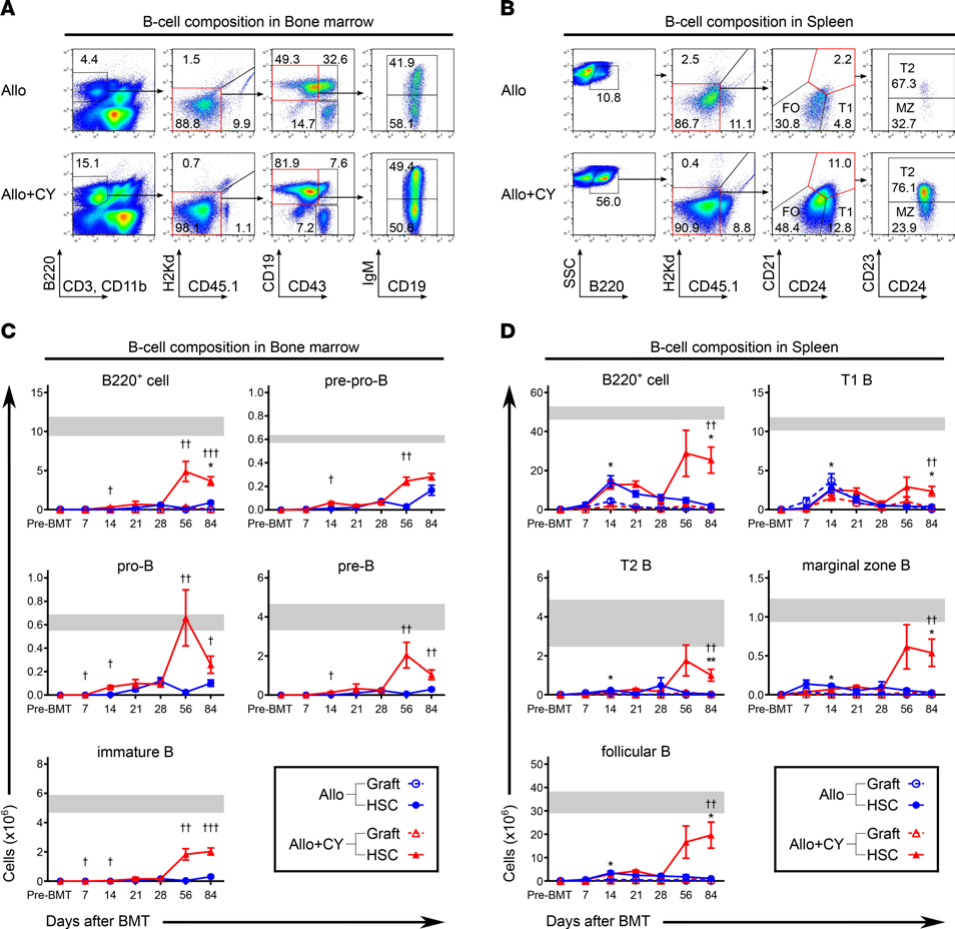
PTCy is associated with an increase in B cell progenitors in the bone marrow and mature B cells in the spleen after BMT. Lethally irradiated (10 Gy) BDF1 recipients (H2K^b/d^CD45.2^+^) received transplants of 5 × 10^6^ Ly 5.1 B6 (H2K^b/b^CD45.1^+^) splenocytes and 5 × 10^6^ B6 (H2K^b/b^CD45.2^+^) TCD-BM cells. All recipient mice were injected intraperitoneally with 50 mg/kg cyclophosphamide or vehicle on day 3 after allogeneic BMT (vehicle-treated, *n* = 30, and PTCy-treated, *n* = 32). (**A** and **B**) Representative flow cytometry plots identifying B220^+^ cell subsets and chimerism in the bone marrow (**A**) and spleen (**B**) 56 days after allogeneic BMT. (**C**) Kinetics of graft- and HSC-derived B220^+^ cells and HSC-derived pre-pro-B, pro-B, pre-B, and immature B cell recovery in the bone marrow after allogeneic BMT. (**D**) Kinetics of graft- and HSC-derived B220^+^, T1 B, T2 B, marginal zone B, and follicular B cell recovery in the spleen after allogeneic BMT. * and ^†^ (**C** and **D**) indicate the comparison between graft-derived B cells in the vehicle-treated group versus those in the PTCy-treated group and HSC-derived B cells in the vehicle-treated group versus those in the PTCy-treated group, respectively. Gray bars (**C** and **D**) indicate the mean reference values ± SEM of NC (*n* = 3). Graft- and HSC-derived cells were defined as H2Kd^−^CD45.1^+^ and H2Kd^−^CD45.1^−^ gated cells, respectively, using flow cytometry. Data from 2 independent experiments were combined and expressed as the mean ± SEM. *P* values were determined using the Mann-Whitney *U* test. *,^†^*P* < 0.05, **,^††^*P* < 0.01, ^†††^*P* < 0.001.

To evaluate the effect of the decrease in graft-derived T cells caused by PTCy on B cell reconstitution after allogeneic BMT, we first assessed the development of B cell progenitors in the bone marrow. Chimerism analysis using flow cytometry revealed that HSC-derived B220^+^ cells were predominant in the bone marrow and spleen during the early phase after allogeneic BMT (Supplemental Figure 4). Nearly all the graft-derived B cells decreased in the bone marrow and spleen immediately after BMT in both the PTCy- and vehicle-treated groups (Figure 3, C and D). The number of HSC-derived B cell progenitors in the PTCy-treated group increased in the late phase after BMT and was significantly higher than that in the vehicle-treated group (Figure 3, A and C). The B cells that migrated from the bone marrow after allogeneic BMT were also analyzed (Figure 3, B and D). The numbers of HSC-derived T1 B, T2 B, marginal zone B, and follicular B cells were significantly higher in the PTCy-treated group than in the vehicle-treated group. Moreover, the PTCy-treated mice had a higher proportion of HSC-derived B cells than vehicle-treated mice during the late phase after BMT (Supplemental Figure 3).

### Decreased BAFF levels and germinal center B cells and increased IL-10–producing regulatory B cells are accompanied by B lymphopoiesis promotion after PTCy.

**Figure 4:**
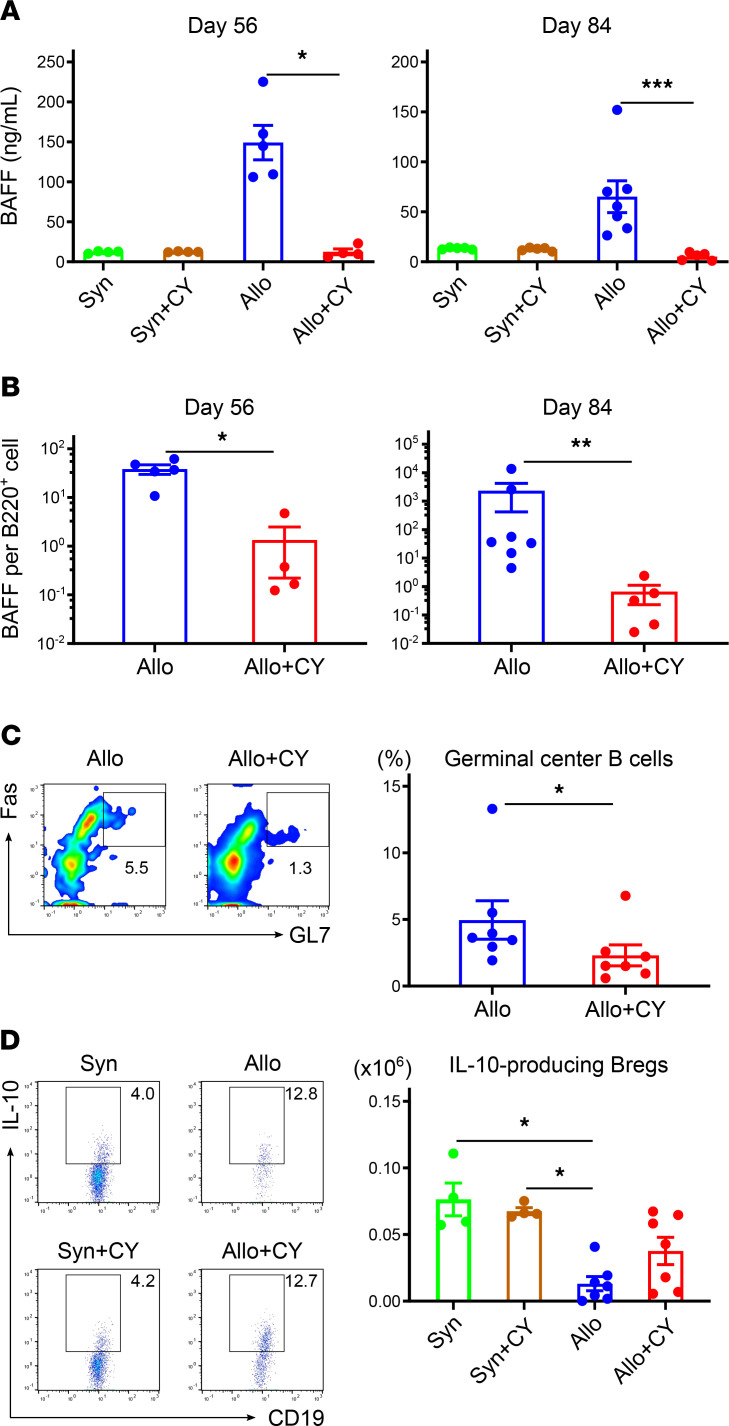
Decreased BAFF levels and germinal center B cells and increased IL-10–producing regulatory B cells are accompanied by B lymphopoiesis promotion after PTCy. Lethally irradiated (10 Gy) BDF1 recipients (H2K^b/d^CD45.2^+^) received transplants of 5 × 10^6^ Ly 5.1 B6 (H2K^b/b^CD45.1^+^) splenocytes and 5 × 10^6^ B6 (H2K^b/b^CD45.2^+^) TCD-BM cells. The syngeneic group was administered the same numbers of splenocytes and TCD-BM cells from the BDF1 mice. All the recipient mice were injected intraperitoneally with 50 mg/kg cyclophosphamide or vehicle on day 3 after BMT. (**A**) Serum levels of BAFF on days 56 (syngeneic/vehicle-treated, *n* = 4; syngeneic/PTCy-treated, *n* = 4; allogeneic/vehicle-treated, *n* = 5; and allogeneic/PTCy-treated, *n* = 4) and 84 (syngeneic/vehicle-treated, *n* = 5; syngeneic/PTCy-treated, *n* = 5; allogeneic/vehicle-treated, *n* = 7; and allogeneic/PTCy-treated, *n* = 5) after BMT (data derived from 2 independent experiments). (**B**) BAFF per HSC-derived B220^+^ cell on day 56 (allogeneic/vehicle-treated, *n* = 5, and allogeneic/PTCy-treated, *n* = 4) and 84 (allogeneic/vehicle-treated, *n* = 7, and allogeneic/PTCy-treated, *n* = 5) after allogeneic BMT (data derived from 2 independent experiments). (**C**) Representative flow cytometry plots identifying GC B (B220^+^GL7^+^Fas^+^) cells and the frequency of GC B cells in the lymph nodes on day 56 after allogeneic BMT (allogeneic/vehicle-treated, *n* = 7; allogeneic/PTCy-treated, *n* = 7; and data derived from 2 independent experiments). (**D**) Representative flow cytometry plots identifying IL-10–producing Bregs in splenic marginal zone B cell (CD19^+^CD21^hi^CD23^−^CD24^int^) population, and the number of Bregs in marginal zone B cell population on day 56 following BMT (syngeneic/vehicle-treated, *n* = 4; syngeneic/PTCy-treated, *n* = 4; allogeneic/vehicle-treated, *n* = 7; allogeneic/PTCy-treated, *n* = 7; and data derived from 1 experiment). Bregs in the syngeneic and allogeneic groups were defined as CD19^+^IL-10^+^H2Kd^+^CD45.1^−^ and CD19^+^IL-10^+^H2Kd^−^CD45.1^−^ gated cells, respectively. Data are expressed as the mean ± SEM. *P* values were determined using the Kruskal-Wallis test (**A** and **D**) and the Mann-Whitney *U* test (**B** and **C**). *P < 0.05, **P < 0.01, ***P < 0.001. GC, germinal center; Breg, regulatory B cell.

We examined changes in serum BAFF levels after BMT using an enzyme-linked immunosorbent assay (ELISA). On days 56 and 84 after BMT, BAFF levels in the allogeneic group without PTCy treatment were significantly higher than those in the allogeneic group with PTCy (Figure 4A). HSC-derived B cell recovery was delayed in the former group (Figure 3D), resulting in excessive BAFF for each B cell type (Figure 4B). Murine studies have demonstrated that germinal center (GC) B cells are required for the development of chronic GVHD (15, 16) whereas donor-derived IL-10–producing Bregs play a suppressive role in the development of chronic GVHD (45). We then assessed the impact of improved B lymphopoiesis on the development of GC B cells and the reconstitution of Bregs after BMT. The PTCy-treated group exhibited a significant reduction in the frequency of GC B cells in the lymph nodes at 56 days after allogeneic BMT (Figure 4C). Although the percentage of Bregs in marginal zone B cells was comparable between the allogeneic groups with and without PTCy treatment (Figure 4D), the recovery of marginal zone B cells was greater in the PTCy-treated group than in the vehicle-treated group (Figure 3D). This resulted in an increase in the number of Bregs in the former group compared with that in the latter group (Figure 4D).

### PTCy is associated with a decrease in chronic GVHD-specific pathologic scores in the late phase following BMT.

**Figure 5:**
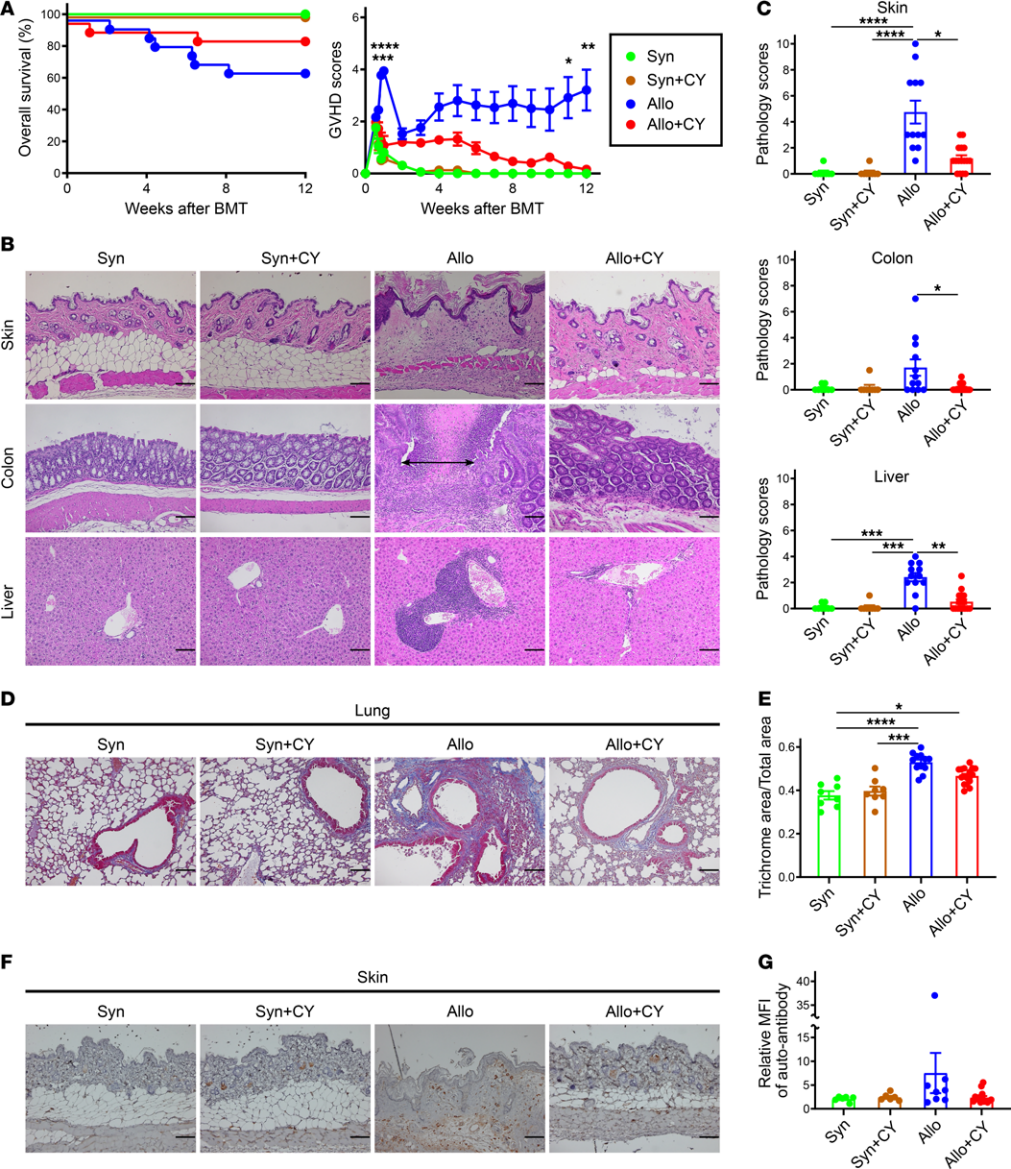
PTCy is associated with a decrease in chronic GVHD-specific pathologic scores in the late phase following BMT. Lethally irradiated (10 Gy) BDF1 recipients (H2K^b/d^CD45.2^+^) received transplants of 5 × 10^6^ splenocytes and 5 × 10^6^ TCD-BM cells from B6 mice (H2K^b/b^CD45.2^+^). The syngeneic group was administered equal amounts of splenocytes and TCD-BM cells from BDF1 mice. All recipient mice were injected intraperitoneally with 50 mg/kg cyclophosphamide or vehicle on day 3 after BMT. All animals were monitored daily for survival, and GVHD scores were monitored from days 4 to 7 and once a week from day 14. (**A**) Kaplan-Meier survival curve and mean GVHD scores of recipient mice in the syngeneic/vehicle-treated (*n* = 8), syngeneic/PTCy-treated (*n* = 8), allogeneic/vehicle-treated (*n* = 18), and allogeneic/PTCy-treated (*n* = 18) groups (GVHD scores: allogeneic/vehicle-treated versus allogeneic/PTCy-treated, *P* < 0.05 day 77, *P* < 0.01 days 5 and 84, *P* < 0.001 day 6, *P* < 0.0001 day 7). (**B**–**F**) Recipient mice were euthanized, and the skin, colon, liver, and lungs were harvested 12 weeks after BMT (syngeneic/vehicle-treated, *n* = 8; syngeneic/PTCy-treated, *n* = 8; allogeneic/vehicle-treated, *n* = 12; and allogeneic/PTCy-treated, *n* = 16). Histopathological analysis of the skin, colon, liver, and lungs was performed. (**B**) Representative images of the skin, colon, and liver from the recipient mice stained with H&E (scale bar = 100 μm, original magnification, ×200). Double-headed arrows indicate mucosal ulcerations. (**C**) GVHD pathological scores for the skin, colon, and liver. (**D**) Representative images of Masson’s trichrome staining of lung (scale bar = 100 μm, original magnification, ×200). Collagen (blue staining) was quantified (**E**). (**F**) Representative images of skin from recipient mice with IgG immunostaining (scale bar = 100 μm, original magnification, ×200). (**G**) The production of anti-recipient IgG per total IgG in serum at 12 weeks after BMT in syngeneic/vehicle-treated (*n* = 6), syngeneic/PTCy-treated (*n* = 6), allogeneic/vehicle-treated (*n* = 8), and allogeneic/PTCy-treated (*n* = 14) groups. Data from 2 independent experiments were combined and expressed as the mean ± SEM. Kruskal-Wallis test. *P < 0.05, **P < 0.01, ***P < 0.001, ****P < 0.0001.

We evaluated the effects of PTCy on long-term survival and GVHD features of the recipients. Lethally irradiated BDF1 mice were transplanted with splenocytes and TCD-BM cells from B6 mice on day 0, then treated with cyclophosphamide on day 3. Syngeneic transplantation was performed as a control. The survival rate of the allogeneic group without PTCy was lower than that of the allogeneic group with PTCy (*P* = 0.11) (Figure 5A). The GVHD scores in the first week posttransplant were significantly higher in the allogeneic vehicle-treated group than in the allogeneic PTCy-treated group (Figure 5A), suggesting that PTCy efficiently suppresses acute GVHD, as previously reported (36, 46). Tissue damage in the late phase was evaluated, and the recipients were anesthetized and euthanized 12 weeks after BMT to harvest skin, colon, liver, and lung samples. Histological sections of the skin from allogeneic recipients without PTCy treatment exhibited standard pathological features of cutaneous GVHD, including dermal fibrosis, fat atrophy, inflammation, and follicular dropout, similar to scleroderma. In addition, the recipient mice showed histopathological evidence of gastrointestinal and hepatic GVHD, such as inflammatory cell infiltration in the lamina propria and portal region (Figure 5B). Pathological scores were significantly higher in the allogeneic group without PTCy treatment than in the allogeneic group (Figure 5C). Recipients in the allogeneic group showed significantly increased collagen deposition around the bronchioles and blood vessels of the lungs compared with those in the syngeneic groups, and PTCy treatment reduced collagen deposition in the lungs of the allogeneic group (Figure 5, D and E). Moreover, we observed a marked infiltration of IgG-positive plasma cells with abundant cytoplasm and an eccentric nucleus into the dermis and subcutaneous fat layer in the allogeneic vehicle-treated group (Figure 5F and Supplemental Figure 5). The production of anti-recipient–specific IgG in the serum increased in the allogeneic group without PTCy treatment compared with that in the other groups (Figure 5G).

### Administration of cyclosporine A or rapamycin is not associated with a decrease in graft-derived effector T cells in bone marrow early after BMT or with an increase in HSC-derived T and B cells in the spleen in the later period.

**Figure 6:**
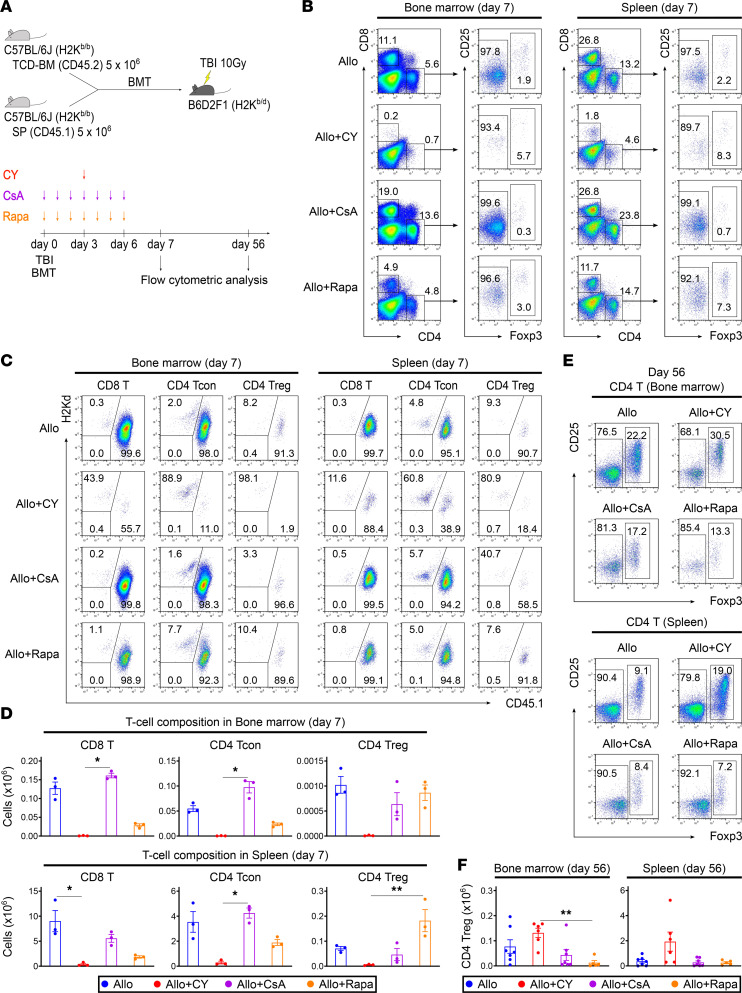
Administration of CsA or Rapa is not associated with a decrease in graft-derived effector T cells in bone marrow early after BMT or with an increase in HSC-derived T cells in the spleen in the later period. (**A**) Lethally irradiated (10 Gy) BDF1 recipients (H2K^b/d^CD45.2^+^) received transplants of 5 × 10^6^ Ly 5.1 B6 (H2K^b/b^CD45.1^+^) splenocytes with 5 × 10^6^ B6 (H2K^b/b^CD45.2^+^) TCD-BM cells. From days 0 to 6 after allogeneic BMT, the recipient mice in the PTCy-treated group were injected intraperitoneally with cyclophosphamide (50 mg/kg) on day 3 and vehicle on the other days. In CsA-treated, Rapa-treated, and vehicle-treated groups, CsA (25 mg/kg), Rapa (0.5 mg/kg), and vehicle were administered for 7 days, respectively. Animals were euthanized on days 7 and 56 after allogeneic BMT to harvest bone marrow and spleens (vehicle-treated, *n* = 10; PTCy-treated, *n* = 9; CsA-treated, *n* = 10; and Rapa-treated, *n* = 8). (**B** and **C**) Representative flow cytometry plots identifying CD4^+^ and CD8^+^ T cell subsets (**B**) and chimerism (**C**) in the bone marrow and spleen on day 7 after allogeneic BMT. (**D**) Numbers of graft-derived CD8^+^ T cells, CD4^+^ Tcons, and CD4^+^ Tregs in the bone marrow and spleen on day 7 following allogeneic BMT (data derived from 1 experiment). (**E**) Representative flow cytometry plots identifying CD4^+^ Tregs in the bone marrow and spleen on day 56 after allogeneic BMT. (**F**) Numbers of HSC-derived Tregs in the bone marrow and spleen on day 56 after allogeneic BMT (data derived from 2 independent experiments). Graft-derived and HSC-derived cells were defined as H2Kd^−^CD45.1^+^ and H2Kd^−^CD45.1^−^ gated cells using flow cytometry, respectively. Data are expressed as the mean ± SEM. *P* values were determined using the Kruskal-Wallis test. *P < 0.05, **P < 0.01.

**Figure 7:**
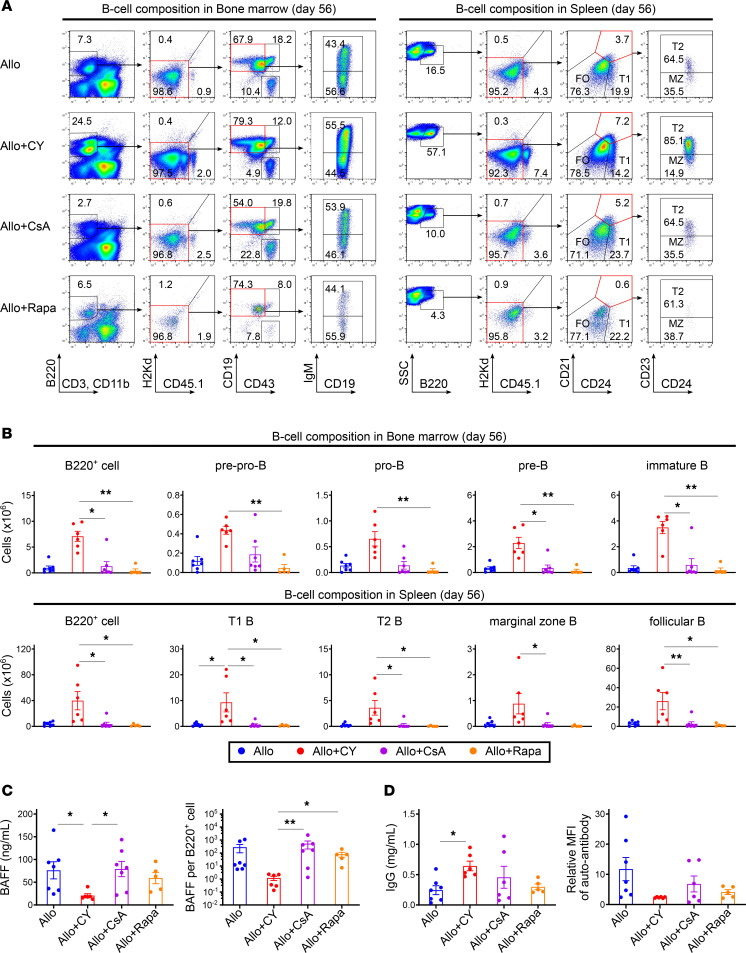
PTCy, but not CsA or Rapa, specifically promotes B lymphopoiesis after allogeneic BMT. Lethally irradiated (10 Gy) BDF1 recipients (H2K^b/d^CD45.2^+^) received transplants of 5 × 10^6^ Ly 5.1 B6 (H2K^b/b^CD45.1^+^) splenocytes and 5 × 10^6^ B6 (H2K^b/b^CD45.2^+^) TCD-BM cells. From days 0 to 6 after allogeneic BMT, recipient mice in the PTCy-treated group were injected intraperitoneally with cyclophosphamide (50 mg/kg) on day 3 and vehicle on the other days. In the CsA-treated, Rapa-treated, and vehicle-treated groups, CsA (25 mg/kg), Rapa (0.5 mg/kg), and vehicle were administered for 7 days, respectively. (**A**) Representative flow cytometry plots identifying B220^+^ cell subsets and chimerism in the bone marrow and spleen 56 days after allogeneic BMT. (**B**) The numbers of HSC-derived B220^+^, pre-pro-B, pro-B, pre-B, and immature B cells in the bone marrow and B220^+^, T1 B, T2 B, marginal zone B, and follicular B cells in the spleen on day 56 after allogeneic BMT (vehicle-treated, *n* = 7; PTCy-treated, *n* = 6; CsA-treated, *n* = 7; and Rapa-treated, *n* = 5). HSC-derived cells were defined as H2Kd^−^CD45.1^−^ gated cells using flow cytometry. (**C**) The serum levels of BAFF and BAFF per HSC-derived B220^+^ cell 56 days after allogeneic BMT (vehicle-treated, *n* = 7; PTCy-treated, *n* = 6; CsA-treated, *n* = 7; and Rapa-treated, *n* = 5). (**D**) The total IgG levels and production of anti-recipient IgG per total IgG in the serum on day 56 after allogeneic BMT (vehicle-treated, *n* = 7; PTCy-treated, *n* = 6; CsA-treated, *n* = 6; and Rapa-treated, *n* = 5). The data from 2 independent experiments were combined and expressed as the mean ± SEM. The *P* values were determined using the Kruskal-Wallis test. *P < 0.05, **P < 0.01.

We subsequently investigated the effects of other immunosuppressants, such as cyclosporine A (CsA) and rapamycin (Rapa), on the restoration of B lymphopoiesis after allogeneic BMT. As an experimental transplantation model, lethally irradiated BDF1 mice were transplanted with splenocytes from Ly 5.1 B6 mice and TCD-BM cells from B6 mice on day 0; cyclophosphamide was administered on day 3, CsA from days 0 to 6, or Rapa from days 0 to 6 (Figure 6A). Rapa partially suppressed the increase in graft-derived effector T cells in the bone marrow and spleen 7 days after BMT, whereas PTCy was associated with a much greater reduction in alloreactive effector T cells than CsA or Rapa (Figure 6, B–D). On day 56 after allogeneic BMT, the recovery of HSC-derived B cell progenitors, mature B cells, and CD4^+^ Tregs was significantly delayed in the CsA- and Rapa-treated groups, whereas it was promoted in the PTCy group (Figure 6, E and F, and Figure 7, A and B). BAFF levels in the CsA- and Rapa-treated groups were higher than those in the PTCy-treated group (Figure 7C). Moreover, the CsA- or Rapa-treated groups exhibited lower total IgG levels but higher production of anti-recipient–specific IgG in the serum than the PTCy-treated group (Figure 7D).

### Depletion of graft-derived Tregs results in complete B cell deficiency; however, PTCy successfully restores donor B cell development and maintenance.

**Figure 8:**
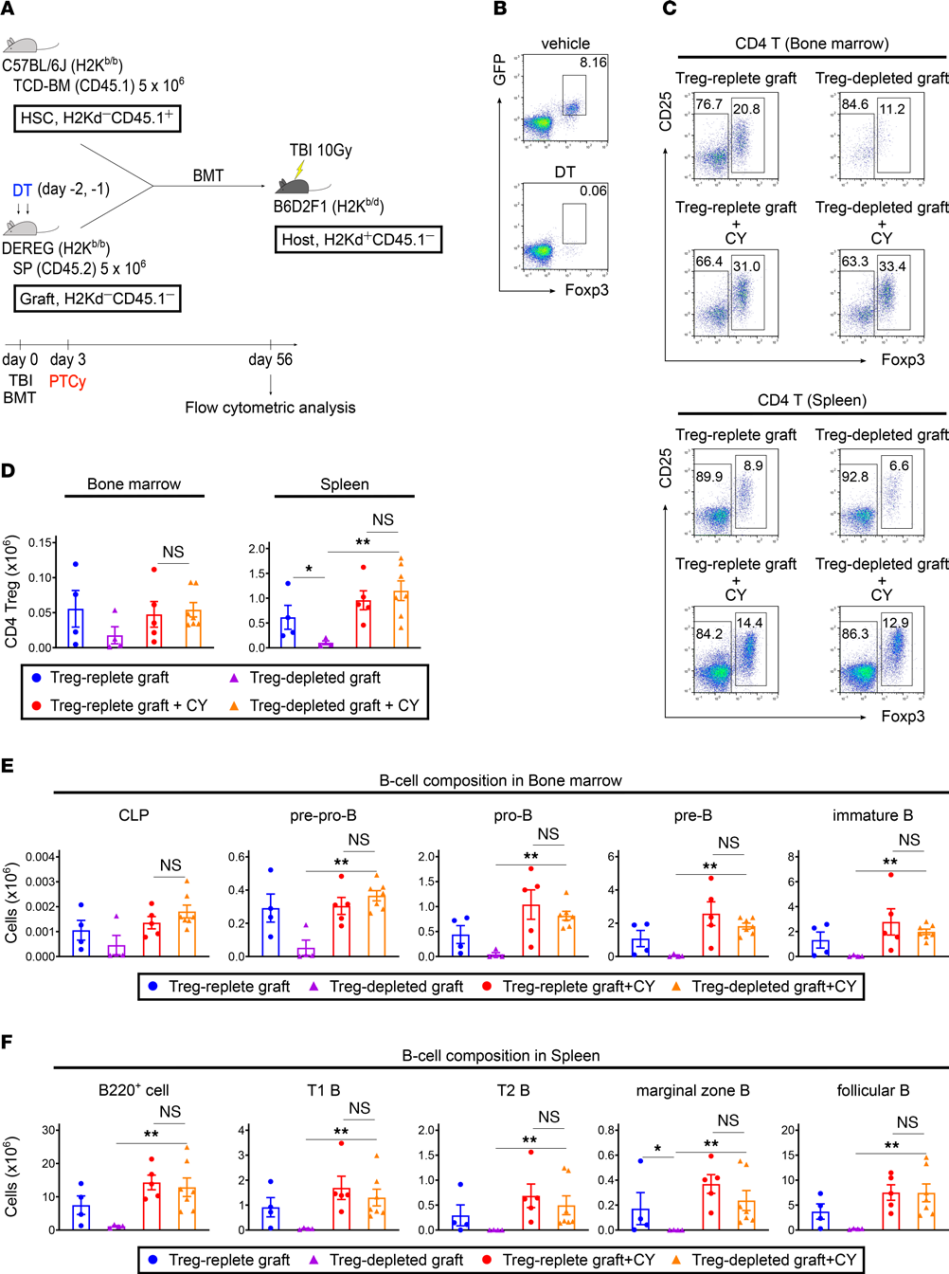
Depletion of graft-derived Tregs results in complete B cell deficiency; however, PTCy successfully restores donor B cell development and maintenance. (**A**) DEREG mice (H2K^b/b^CD45.2^+^) were injected intraperitoneally with 50 ng/g of DT or vehicle on days –2 and –1. Then, lethally irradiated (10 Gy) BDF1 recipients (H2K^b/d^CD45.2^+^) received transplants of 5 × 10^6^ DEREG splenocytes with 5 × 10^6^ Ly 5.1 B6 (H2K^b/b^CD45.1^+^) TCD-BM cells. All recipient mice were injected intraperitoneally with 50 mg/kg of cyclophosphamide or vehicle on day 3 after allogeneic BMT. All animals were monitored daily for survival, and GVHD scores were monitored from days 4 to 7 and once a week from day 14. On day 56, all animals were euthanized following allogeneic BMT to harvest bone marrow and spleens (DT-untreated/PTCy-untreated, *n* = 4; DT-treated/PTCy-untreated, *n* = 4; DT-untreated/PTCy-treated, *n* = 5; and DT-treated/PTCy-treated, *n* = 7). (**B**) Representative flow cytometry plots identifying GFP^+^Foxp3^+^ cells gated on CD4^+^ T cells in the spleen of DT-untreated and -treated DEREG mice. (**C**) Representative flow cytometry plots identifying CD4^+^ Tregs in the bone marrow and spleen on day 56 after allogeneic BMT. (**D**) The numbers of HSC-derived Tregs in the bone marrow and spleen on day 56 after allogeneic BMT. (**E**) The numbers of HSC-derived CLP, pre-pro-B, pro-B, pre-B, and immature B cells in the bone marrow on day 56 after allogeneic BMT. (**F**) The numbers of HSC-derived B220^+^, T1 B, T2 B, marginal zone B, and follicular B cells in the spleen on day 56 after allogeneic BMT. The HSC-derived cells were defined as H2Kd^−^CD45.1^−^ gated cells using flow cytometry. Data were obtained from 1 experiment and expressed as the mean ± SEM. *P* values were determined using the Kruskal-Wallis test. *P < 0.05, **P < 0.01. DEREG, B6-Tg (Foxp3-DTR/EGFP) 23.2Spar/Mmjax.

To evaluate the effects of graft-derived Tregs on donor B cell reconstitution, we used B6-Tg (Foxp3-DTR/EGFP) 23.2Spar/Mmjax (DEREG) mice as our BMT model. DEREG mice express a fusion protein of the diphtheria toxin (DT) receptor and enhanced green fluorescent protein under the control of the *Foxp3* gene locus (47). Previous studies demonstrated that Foxp3^+^ Tregs can be selectively depleted by DT administration in DEREG mice (13, 47, 48). We transplanted TCD-BM cells from Ly 5.1 B6 mice and splenocytes from DT-treated DEREG mice into lethally irradiated BDF1 mice (Figure 8A). In this experiment, splenocytes from DEREG mice and TCD-BM cells from Ly 5.1 B6 mice were determined as “Graft” and “HSC,” respectively. DEREG mice received DT on days –2 and –1. We did not detect CD4^+^GFP^+^Foxp3^+^ cells in the spleens of DT-treated DEREG mice (Figure 8B). We verified a substantial decrease in the number of HSC-derived Tregs in PTCy-untreated recipients that received Treg-depleted grafts compared with those that received Treg-replete grafts on day 56 after BMT, whereas Tregs increased in PTCy-treated recipients that received Treg-depleted grafts (Figure 8, C and D). When graft-derived Tregs were depleted, B cell reconstitution was critically impaired after allogeneic BMT in PTCy-untreated recipients; however, PTCy treatment restored B cell development and maintenance even after transplantation of Treg-depleted grafts (Figure 8, E and F, and Supplemental Figure 6A).

### Depletion of HSC-derived Tregs also results in complete B cell deficiency, and PTCy cannot restore donor B cell development.

**Figure 9:**
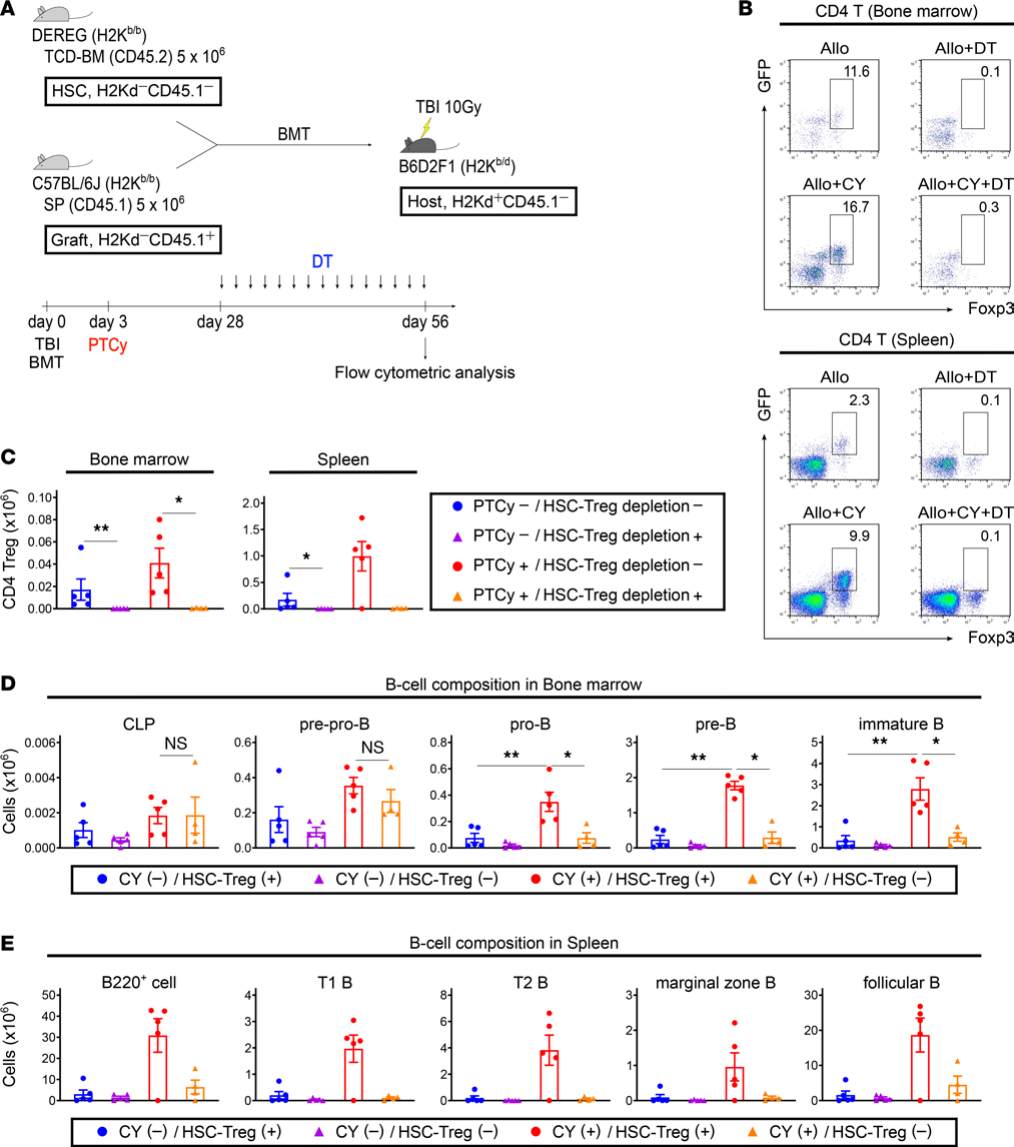
Depletion of HSC-derived Tregs also results in complete B cell deficiency, and PTCy cannot restore donor B cell development. (**A**) Lethally irradiated (10 Gy) BDF1 recipients (H2K^b/d^CD45.2^+^) received transplants of 5 × 10^6^ Ly 5.1 B6 (H2K^b/b^CD45.1^+^) splenocytes with 5 × 10^6^ DEREG (H2K^b/b^CD45.2^+^) TCD-BM cells. All recipient mice were injected intraperitoneally with 50 mg/kg of cyclophosphamide or vehicle on day 3 after allogeneic BMT, then injected intraperitoneally with 10 ng/g of DT or vehicle every other day from days 28 to 56 after allogeneic BMT. All animals were monitored daily for survival, and GVHD scores were monitored from days 4 to 7 and once a week from day 14. On day 56 after allogeneic BMT, all animals were euthanized to harvest bone marrow and spleens (PTCy-untreated/DT-untreated, *n* = 5; PTCy-untreated/DT-treated, *n* = 5; PTCy-treated/DT-untreated, *n* = 5; and PTCy-treated/DT-treated, *n* = 4). (**B**) Representative flow cytometry plots identifying CD4^+^ Tregs in the bone marrow and spleen on day 56 after allogeneic BMT. (**C**) The numbers of HSC-derived Tregs in the bone marrow and spleen on day 56 after allogeneic BMT. (**D**) The numbers of HSC-derived CLP, pre-pro-B, pro-B, pre-B, and immature B cells in the bone marrow on day 56 after allogeneic BMT. (**E**) The numbers of HSC-derived B220^+^, T1 B, T2 B, marginal zone B, and follicular B cells in the spleen on day 56 after allogeneic BMT. HSC-derived Tregs and B cells were defined as CD4^+^GFP^+^Foxp3^+^ and B220^+^H2Kd^−^CD45.1^−^ gated cells using flow cytometry, respectively. Data were obtained from 1 experiment and expressed as the mean ± SEM. *P* values were determined using the Kruskal-Wallis test. *P < 0.05, **P < 0.01.

A mouse BMT model in which HSC-derived Tregs could be selectively depleted in vivo was used to evaluate the effects of HSC-derived Tregs on donor B cell reconstitution (Figure 9A). Recipients transplanted with splenocytes from Ly 5.1 B6 mice and TCD-BM cells from DEREG mice received DT every alternate day from days 28 to 56 after BMT. In this experiment, splenocytes from Ly 5.1 B6 mice and TCD-BM cells from DEREG mice were determined as “Graft” and “HSC,” respectively. We observed a significant decrease in the number of HSC-derived Tregs in the bone marrow of recipients receiving DT compared with those receiving vehicle on day 56 following BMT (Figure 9, B and C). In the PTCy-treated groups, the numbers of CLP and pre-pro-B cells were maintained regardless of HSC-derived Treg depletion; however, cellular differentiation from pre-pro-B cells to further maturational stages was significantly impaired in recipients in which HSC-derived Tregs were depleted (Figure 9, D and E, and Supplemental Figure 6B).

## Discussion

Long-term prospective analyses of patients undergoing allogeneic HSCT have revealed that the development of chronic GVHD is associated with the impaired reconstitution of Tregs and B cells in the peripheral blood (7, 20). However, the sequential immune process of hematopoietic recovery in bone marrow after HSCT remains unclear. Our data demonstrate that the reduction in alloreactive effector T cells in the bone marrow early after BMT could preserve bone marrow hematopoietic function, which contributes to the early restoration of posttransplant B cell development after PTCy. We also demonstrated that the reconstitution of HSC-derived Tregs, but not graft-derived Tregs, plays an essential role in maintaining intra–bone marrow B cell development. These results may help elucidate the underlying immune mechanisms by which PTCy prevents chronic GVHD as well as acute GVHD. We previously reported that PTCy ameliorated acute GVHD in a dose-dependent manner and that 50 mg/kg PTCy alone was sufficient to prevent GVHD, whereas this dose of PTCy reduced graft-versus-leukemia (GVL) activity in the MHC-haploidentical BMT system (49). While a previous study evaluated GVL activity and acute GVHD, the current study primarily addressed clinical symptoms and immune abnormalities related to chronic GVHD and the impact of PTCy using fewer donor T cells in the same mouse BMT system.

We first examined the reconstitution of lymphocyte pools in the bone marrow as a primary lymphoid organ and in the spleen as a peripheral lymphoid organ (hereinafter referred to as “periphery”) during the first month after allogeneic BMT (Figure 1 and Supplemental Figure 1). In the spleen, a transient surge in the number of graft-derived effector T cells peaked on day 14; thereafter, the number of cells decreased until day 28 (Figure 1B), as previously reported (50–52). In contrast, in the bone marrow, the number of cells increased approximately 5-fold compared with normal levels immediately after allogeneic BMT and remained elevated until day 28 (Figure 1B). Unlike effector T cells, the number of Tregs and B cells did not increase in the bone marrow or spleen during this period (Figure 1, B, D, and E). These data indicated that the imbalance between graft-derived effector T cells and Tregs in the early phase following allogeneic BMT was more evident and prolonged in the bone marrow than in the periphery.

To assess the impact of T cell subset imbalance in the bone marrow in the first month on subsequent bone marrow hematopoietic function, we sequentially measured the recovery of peripheral blood cells and bone marrow cellularity until 3 months following allogeneic BMT (Figure 2B and Supplemental Figure 2). In recipients receiving BMT without PTCy treatment, the number of hematopoietic cells did not recover to the normal range until 3 months posttransplantation. However, hematopoietic cells recovered 2 months after BMT in recipients treated with PTCy (Figure 2B and Supplemental Figure 2). These data indicate that the imbalance of T cell subsets in the bone marrow could critically affect subsequent hematopoiesis and that PTCy could help restore normal hematopoiesis.

To investigate the protective effects of PTCy treatment on bone marrow hematopoietic function, we analyzed long-term T and B lymphopoiesis 3 months after BMT (Figures 2 and 3). The analysis revealed that PTCy inhibited the increase in graft-derived effector T cells in the bone marrow during the first month (Figure 2E and Supplemental Figure 3). Thereafter, PTCy significantly increased the number of CLPs, double-positive thymocytes, and mature T cell subsets that differentiated from the donor HSCs (Figure 2, E–G). In the vehicle-treated group, B cell recovery was barely observed in the first 3 months except for a transient increase in mature B cells that appeared to have differentiated from B cell progenitors in the transplanted graft. In contrast, in the PTCy-treated group, a rapid increase in the number of pro-B cells was observed in the bone marrow on day 56, followed by a stable increase in mature B cells in the spleen (Figure 3, C and D, and Supplemental Figure 3). These data indicate that PTCy suppressed the initial increase in alloreactive effector T cells in the bone marrow, suggesting that the control of graft-derived effector T cells immediately after transplantation contributes to the preservation of bone marrow hematopoietic function, which promotes the restoration of T and B lymphopoiesis after allogeneic BMT.

In the vehicle-treated group, BAFF levels significantly increased to compensate for impaired B lymphopoiesis, which may be associated with an increase in GC B cells that produce pathological immunoglobulins for chronic GVHD development. In the PTCy-treated group, the BAFF levels were lower than those in the syngeneic groups, which may be associated with decreased differentiation into GC B cells and an increase in the number of Bregs that function in tolerogenic immune responses (Figure 4). These data show that rapid filling of the normal B cell pool after allogeneic BMT could contribute to a well-balanced reconstitution of B cell subsets through a decrease in BAFF concentration. We believe that our results regarding Bregs reflect favorable B cell reconstitution by PTCy. However, the Breg detection method used in the present study required ex vivo cytokine stimulation; therefore, it may not directly evaluate Bregs in vivo. It may be necessary to apply other methods for a more accurate Breg evaluation in the future.

Surviving mice treated without PTCy for a longer period after allogeneic BMT exhibited multiorgan tissue fibrosis, pathological features of chronic GVHD, and ongoing acute GVHD target organ injury (Figure 5, B–E). In addition, IgG-positive plasma cells infiltrated the skin in the allogeneic vehicle-treated group (Figure 5F and Supplemental Figure 5). Serum anti-recipient–specific IgG levels were increased in allogeneic recipients without PTCy treatment (Figure 5G), suggesting a possible role for B cells in the late phase of GVHD pathogenesis in this model. Further development of clinically relevant chronic GVHD models may provide important findings that could lead to long-term clinical applications.

To evaluate whether the promotion of B lymphopoiesis after allogeneic BMT is specific to PTCy, the effects of different pharmacological immunosuppressants on B lymphopoiesis using CsA or Rapa were investigated, which have been used for GVHD prophylaxis (53, 54) (Figures 6 and 7). The analysis showed that Rapa, but not CsA, partially reduced graft-derived effector T cells in the bone marrow without decreasing Tregs (Figure 6, B–D). However, B lymphopoiesis in the late phase after allogeneic BMT was significantly impaired in both the CsA- and Rapa-treated groups compared with that in the PTCy-treated group, in which graft-derived T cells were significantly decreased in the bone marrow (Figure 7, A and B). Additionally, compared with the PTCy-treated group, the BAFF for each B cell was significantly higher, and anti-recipient–specific antibody production increased in the CsA- and Rapa-treated groups (Figure 7, C and D). These data suggest that the promoting effect on posttransplant B lymphopoiesis through the suppression of alloreactive T cell increases in the bone marrow appears to be specific to PTCy treatment rather than to CsA or Rapa treatments. However, the reasons why donor T cells tend to increase in the bone marrow early after BMT and the mechanism by which PTCy suppresses this increase more efficiently than other immunosuppressants remain unclear, and further studies are required. Additionally, in actual clinical situations, CsA or Rapa is administered for longer periods; thus, we anticipate that these agents may have mechanisms to maintain B cell reconstitution other than early bone marrow preservation. Therefore, we plan to perform a comprehensive multiomics analysis of bone marrow samples obtained serially from patients transplanted using various immunosuppressive techniques to further understand the mechanisms of posttransplant B cell recovery.

Finally, we assessed the effect of Treg reconstitution on B lymphopoiesis after PTCy treatment. After BMT, the Treg population consists of graft- and HSC-derived Tregs. Using the DEREG system, we selectively depleted each Treg to identify which Treg population was important for maintaining long-term B cell homeostasis after BMT (Figures 8 and 9 and Supplemental Figure 6). Our data demonstrated that the negative impact of graft-derived Treg depletion on B cell homeostasis could be largely mitigated by PTCy (Figure 8 and Supplemental Figure 6A); however, HSC-derived Treg depletion resulted in the failure of B cell reconstitution even after PTCy treatment (Figure 9 and Supplemental Figure 6B), indicating the essential role of HSC-derived Tregs in long-lasting B cell–mediated tolerance in PTCy-based allogeneic BMT.

Previous studies have reported that IL-7 plays an important role in maintaining the proper expression levels of early B cell factors for further differentiation at the pre-pro-B cell stage (55, 56). A recent study demonstrated that Treg depletion reduced IL-7 production by perivascular stromal cells in the bone marrow and prevented pre-pro-B cells from differentiating into mature stages under physiological conditions (13). In allogeneic BMT settings, studies have revealed that donor Treg depletion induces severe GVHD (12, 36), an increase in Tregs in the donor T cell inoculum restores the differentiation of pre-pro-B cells to the next stage (14), and the adoptive transfer of graft-derived Tregs attenuates cutaneous chronic GVHD (12). In the present study, host-derived Tregs decreased in the bone marrow soon after transplantation, and graft-derived Tregs did not increase in the early phase following allogeneic BMT. After the first month, HSC-derived Tregs increased significantly and constituted a major component of Tregs in the bone marrow of the PTCy-treated group (Figure 1B; Figure 2, C–E; and Supplemental Figures 1 and 4). Notably, we found that an increase in HSC-derived Tregs is essential for the differentiation of pre-pro-B cells into pro-B cells in the bone marrow. Our results suggest that HSC-derived Tregs may contribute to the preservation of cytokine-producing stromal cells that support favorable B cell development, which can promote immune tolerance after PTCy-based BMT.

In general, transitional B cells, which are immature B cells that have recently emigrated from the bone marrow, develop into mature B cells such as marginal zone and follicular B cells via BCR-mediated negative selection (57). Follicular B cells transport immune complexes formed after antigen presentation by macrophages into follicles in secondary lymphoid organs, initiating a GC response (58, 59). Although physiological levels of BAFF play an important role in maintaining B cell homeostasis at a steady state (60), increased BAFF promotes the survival of self-reactive B cells, which are normally eliminated during the maturation stage (61). In a mouse model of chronic GVHD, excess BAFF and alloantigen synergistically increased BCR-activated B cells (24). In patients with chronic GVHD, high BAFF levels are associated with an increase in activated B cells in cases of persistent BAFF elevation due to B lymphopenia after HSCT (20). Our results suggested that the early increase in transitional and mature B cells after PTCy treatment led to a reduction in BAFF levels, which suppressed excessive differentiation into GC B cells with increasing Bregs. Examining the role of Tregs in GC maturation in the lymph nodes, a previous study reported that adoptive transfer of donor Tregs allowed their infiltration into the GC and suppressed B cell activation, resulting in the amelioration of chronic GVHD (62). As presented in this study, early recovery of HSC-derived Tregs by controlling graft-derived T cells may not only support bone marrow B cell development but also suppress B cell activation in GC in peripheral lymph nodes.

In conclusion, the current study depicted a basic framework for posttransplant B cell reconstitution that could influence chronic GVHD development in the bone marrow and spleen during the 3 months following allogeneic BMT. PTCy was observed to preserve bone marrow hematopoietic function by preventing graft-derived effector T cells from increasing in the bone marrow, which allowed early reignition of B cell development in the bone marrow. HSC-derived Tregs, but not graft-derived Tregs, play an essential role in maintaining favorable intra–bone marrow B cell development and peripheral B cell homeostasis after PTCy, which promotes long-term immune tolerance. Further studies to elucidate the detailed pathophysiology of allogeneic immune reactions against bone marrow niches would facilitate an in-depth understanding of B cell–mediated immunity after BMT.

## Methods

### Mice.

Female B6 (CD45.2, H2K^b/b^) and BDF1 (CD45.2, H2K^b/d^) mice were purchased from SLC. Female Ly 5.1 B6 (CD45.1, H2K^b/b^) mice were purchased from RIKEN BioResource Center. Female DEREG (CD45.2, H2K^b/b^) mice were purchased from Jackson Laboratory. All mice were given food and water ad libitum under specific pathogen–free conditions, and all mice were aged between 8 and 12 weeks, ensuring that the mean body weight in each group was similar.

### BMT.

On day 0, female BDF1 mice were conditioned by lethal irradiation divided into 2 doses (5 Gy each), 6 hours apart. Recipient mice were injected with 5 × 10^6^ splenocytes from Ly 5.1 B6 mice and 5 × 10^6^ TCD-BM cells from B6 mice on day 0 (allogeneic group). T cell depletion from donor bone marrow cells was conducted using anti-CD90.2 MicroBeads and an AutoMACS system (Miltenyi Biotec), according to the manufacturer’s instructions. The syngeneic group was administered the same amount of splenocytes and TCD-BM cells from the BDF1 mice.

### Chimerism analysis.

Lymphocytes in recipients originate from the host or the donor. Donor-derived lymphocytes can be further divided into 2 types: mature cells in donor spleen grafts and newly differentiated cells from donor HSCs. To study the chimerism in each lymphocyte subset in detail, 3 populations of different origins were defined as, in order, “Host,” “Graft,” and “HSC.” Using congenic markers, these cells were defined as H2Kd^+^/CD45.1^−^, H2Kd^−^/CD45.1^+^, and H2Kd^−^/CD45.1^−^ gated cells.

### Posttransplant cyclophosphamide.

Cyclophosphamide (MilliporeSigma) was reconstituted in sterile saline at 5 mg/mL, then administered intraperitoneally at a dose of 50 mg/kg on day 3 after BMT in the PTCy-treated group. In the vehicle-treated group, similar volumes of vehicle were administered intraperitoneally at the same time.

### CsA and Rapa treatments.

CsA (Nacalai Tesque) and Rapa (Santa Cruz Biotechnology) were reconstituted in sterile saline at 2.5 mg/mL and 0.05 mg/mL, respectively. A CsA dosage of 25 mg/kg and a Rapa dosage of 0.5 mg/kg were administered according to a previous study using the C57BL/6-into-BDF1 system (63). Intraperitoneal injections of CsA and Rapa were administered once daily from days 0 to 6 in the CsA-treated and Rapa-treated groups, respectively.

### Assessment of GVHD.

Survival after BMT was monitored daily, and the degree of clinical GVHD was assessed from days 4 to 7 and then weekly using a scoring system for 5 clinical parameters: weight loss, posture, activity, fur texture, and skin integrity (maximum index, 10) as described previously (64).

### Flow cytometry and CBC.

Bone marrow cells were harvested from the tibiae and femurs; whereas lymph nodes were harvested from the mesenteries and inguina of recipient mice. Single-cell suspensions of spleens, bone marrow, thymi, and lymph nodes were incubated with monoclonal antibodies and analyzed using a MACSQuant flow cytometer (Miltenyi Biotec). Data were analyzed using FlowJo software (TreeStar). The detailed methods of staining, including IL-10 intracellular stain, and the mAbs used in this study are shown in the Supplemental Methods. PocH-100iV (Sysmex) was used to conduct CBC.

### ELISA.

Serum samples were collected from the mice on days 56 and 84 after BMT and assayed for soluble BAFF and IgG using an ELISA with commercially available kits (R&D Systems and Bethyl Laboratories, respectively).

### Histopathology.

Skin, colon, liver, and lung specimens from the recipients were fixed in 10% formalin, embedded in paraffin wax, sectioned (4 μm thick), mounted on slides, and stained with hematoxylin and eosin to determine pathology and with Masson’s trichrome to detect collagen deposition. Images were captured using BZ-8100 fluorescence microscope (Keyence). Skin sections were scored by a pathologist based on dermal fibrosis, fat loss, inflammation, epidermal interface changes, and follicular dropout (0–2 for each category, with a maximum score of 10) (65). Colon and liver slides were scored using a semiquantitative scoring system (0.5–4.0 grades), as previously described (66). Collagen deposition in the lung was quantified on trichrome-stained sections as a ratio of blue area to total area using ImageJ software (NIH), as described previously (67).

### Immunohistochemistry.

Formalin-fixed, paraffin-embedded sections (4 μm thick) of the skin were deparaffinized in d-limonene and graded alcohol. For antigen retrieval, the sections were incubated in 1 mM EDTA buffer (pH 8.0) for 20 minutes in a 95°C water bath, followed by incubation with 3% hydrogen peroxide in methanol for 5 minutes at room temperature to block endogenous peroxidase activity. The slides were rinsed with tris(hydroxymethyl)-aminomethane–buffered saline containing 0.1% Tween 20 (Nacalai Tesque) and blocked with goat serum for 1 hour. Subsequently, the sections were incubated with rabbit anti-mouse IgG antibody (Invitrogen 31194) diluted to 1:2,000 overnight at 4°C, followed by incubation with the envision system–labeled polymer, horseradish peroxidase anti-rabbit antibody (DAKOCytomation K4003) for 20 minutes. Finally, the sections were incubated with 3,3-diaminobenzidine and counterstained with hematoxylin.

### Detection of anti-recipient–specific IgG.

Anti-recipient–specific IgG in serum samples was detected via indirect immunofluorescence staining of splenocytes harvested from BDF1 mice using a flow cytometry assay (68). Serum samples were collected from recipient mice and frozen at −80°C. CD19^+^ B cells of BDF1 splenocytes were excluded from the analysis to avoid any confounding effect of antibody binding to cells expressing Fc receptors or B cells expressing membrane immunoglobulin. Splenocytes were added to the recipient serum and incubated at 4°C for 1 hour. FITC-conjugated anti-mouse IgG F(ab′)_2_ (eBioscience 11-4010-82) was then added and incubated for another 30 minutes. The MFI of CD3^+^CD19^−^ cells was used to determine the anti-recipient–specific IgG levels.

### Graft- or HSC-derived Treg depletion.

In the experiment where graft-derived Tregs were depleted, DT (Merck) was diluted in PBS at 5 μg/mL, and DEREG mice in which Tregs were depleted through DT administration (13, 47, 48) were injected intraperitoneally at a dose of 50 ng/g on days –2 and –1. On day 0, female BDF1 mice were conditioned by lethal irradiation divided into 2 doses (5 Gy each), 6 hours apart. Recipient mice were transplanted with 5 × 10^6^ splenocytes from DT-treated DEREG mice and 5 × 10^6^ TCD-BM cells from Ly 5.1 B6 mice. In the experiment in which HSC-derived Tregs were depleted, female BDF1 mice were conditioned by lethal irradiation divided into 2 doses (5 Gy each), 6 hours apart on day 0. Recipient mice were transplanted with 5 × 10^6^ splenocytes from Ly 5.1 B6 mice and 5 × 10^6^ TCD-BM cells from DEREG mice. DT diluted in PBS at 1 μg/mL was administered intraperitoneally at a dose of 10 ng/g every other day from days 28 to 56 after allogeneic BMT.

### Statistics.

The data are presented as mean ± SEM. The Mann-Whitney *U* test was used to assess statistical significance between the allogeneic/vehicle-treated and allogeneic/PTCy-treated groups, and group comparisons among more than 2 groups were performed using the Kruskal-Wallis test. The Kaplan-Meier product limit method was used to determine survival probability, and the log-rank test was applied to compare survival curves. All the tests were 2 tailed, and the results were considered statistically significant at *P* < 0.05. GraphPad Prism 8 software was used for the statistical analyses.

### Study approval.

The study protocols were reviewed and approved by the Animal Care and Use Committee of Okayama University Advanced Science Research Center. All experiments were performed in accordance with the study protocol. The study was conducted in compliance with the ARRIVE guidelines.

## Author contributions

Experiments were designed and performed and the manuscript written by Y Sumii, TK, SI, TF, MI, HS, Y Sando, MN, and Y Meguri. MFN obtained the pathological skin scores. TM, NT, MK, NA, and DE advised on experimental design. Y Maeda supervised laboratory studies and edited the manuscript. KM designed and supervised the study and edited the manuscript.

## Supplementary Material

Supplemental data

## Version 1 04/24/2023

Electronic publication
