# Supplementary material for: Hematopoietic stem cell–derived Tregs are essential for maintaining favorable B cell lymphopoiesis following posttransplant cyclophosphamide
Source: JCI Insight. 2023 Apr 24;8(8):e162180. doi: 10.1172/jci.insight.162180 (PMC10243810; doi:10.1172/jci.insight.162180)
Supplement: Supplemental data [file jciinsight-8-162180-s067.pdf]

Supplemental Methods

For the flow cytometric analysis, cells were first incubated with the following directly conjugated monoclonal antibodies (mAbs) (obtained from eBioscience, San Diego, CA, unless otherwise stated) for 20 min at 4 °C: Pacific Blue-conjugated anti-CD3 (17A2; BioLegend, San Diego, CA); eFluor450-conjugated anti-CD4 (GK 1.5) and anti-CD45R/B220 (RA3-6B2); Brilliant Violet 421-conjugated anti-IL-7R (A7R34; BioLegend); Brilliant Violet 510- or fluorescein isothiocyanate (FITC)-conjugated anti-H2Kd (SF1-1.1; BD Biosciences, San Jose, CA); FITC-conjugated anti-GL7 (GL7; BioLegend); phycoerythrin (PE)-conjugated anti-CD24 (M1/69), anti-CD43 (1B11; BioLegend), and anti-Flt3 (A2F10; BioLegend); PE- or peridinin chlorophyll protein (PerCP)-Cy5.5-conjugated anti-CD45.1 (A20); Brilliant Violet 510- or PE-Cy7-conjugated anti-CD23 (B3B4; BioLegend); PE-Cy7-conjugated anti-CD25 (PC61.5) and anti-c-kit (ACK2; BioLegend); PE-Cy7- or allophycocyanin (APC)-Cy7-conjugated anti-CD19 (6D5; BioLegend, 1D3; BD Biosciences); APC-conjugated anti-CD21/CD35 (7E9; BioLegend), anti-Fas (SA367H8; BioLegend), anti-IgM (RMM-1; BioLegend), and anti-Sca-1 (D7; BioLegend); and APC-eFluor780-conjugated anti-CD8 (53-6.7). For bone marrow B cells and lineage negative cell detection, biotinylated anti-CD3 (17A2), anti-CD11b (M1/70), anti-CD45R/B220 (RA3-6B2), anti-Gr-1 (RB6-8C5), and anti-TER-119 (Ter-119) (all from BioLegend) were used, followed by APC-eFluor780-conjugated streptavidin. To analyze B-cell subsets, Fc receptors were blocked with mouse Fc receptor-specific mAbs (2.4G2; BD Biosciences) before cell surface staining. For Treg detection, cells were processed for intracellular staining using a Foxp3

staining buffer set (eBioscience), and then incubated with APC-conjugated anti-Foxp3 (FJK-16s) for 30 min at 4 °C.

IL-10 analysis using flow cytometry was performed as previously described (1, 2). Briefly, single-cell leukocyte suspensions from the spleens were resuspended (2×10^6 cells/mL) in complete medium [RPMI 1640 medium (Sigma-Aldrich, St. Louis, MO) containing 10% fetal calf serum (FCS; Gibco, Carlsbad, CA), 200 μg/mL penicillin (Gibco), 200 U/mL streptomycin (Gibco), 4 mM L-glutamine (Sigma-Aldrich), and 5×10^{-5} M 2-mercaptoethanol (Sigma-Aldrich)] and stimulated with lipopolysaccharide (LPS; 10 μg/mL, *Escherichia coli* serotype 0111:B4; Sigma-Aldrich), phorbol myristate acetate (PMA; 50 ng/mL; Sigma-Aldrich), ionomycin (500 ng/mL; Sigma-Aldrich), and monensin (2 μM; BD Biosciences) for 5 h at 37 °C. For IL-10 detection, Fc receptors were blocked with the mouse Fc receptor mAb (2.4G2; BD Biosciences), whereas dead cells were detected using a LIVE/DEAD fixable violet dead cell stain kit (Invitrogen, Carlsbad, CA) before cell surface staining. Stained cells were fixed and permeabilized using a Cytofix/Cytoperm kit (BD Biosciences) according to the manufacturer's instructions and then stained with PE-conjugated anti-IL-10 (JES5-16E3; BioLegend). An isotype-matched mAb served as a negative control for IL-10 staining to establish background IL-10 staining levels. The number of splenic IL-10-producing B cells was determined via intracellular staining (2, 3).

References

1. Yanaba K, Bouaziz JD, Haas KM, Poe JC, Fujimoto M, and Tedder TF. A regulatory B cell subset with a unique CD1dhiCD5+ phenotype controls T cell-dependent inflammatory responses. *Immunity.* 2008;28(5):639-50.

2. Matsushita T, and Tedder TF. Identifying regulatory B cells (B10 cells) that produce IL-10 in mice. *Methods Mol Biol.* 2011;677:99-111.

3. Le Huu D, Matsushita T, Jin G, Hamaguchi Y, Hasegawa M, Takehara K, et al. Donor-derived regulatory B cells are important for suppression of murine sclerodermatous chronic graft-versus-host disease. *Blood.* 2013;121(16):3274-83.

Supplemental Figure 1

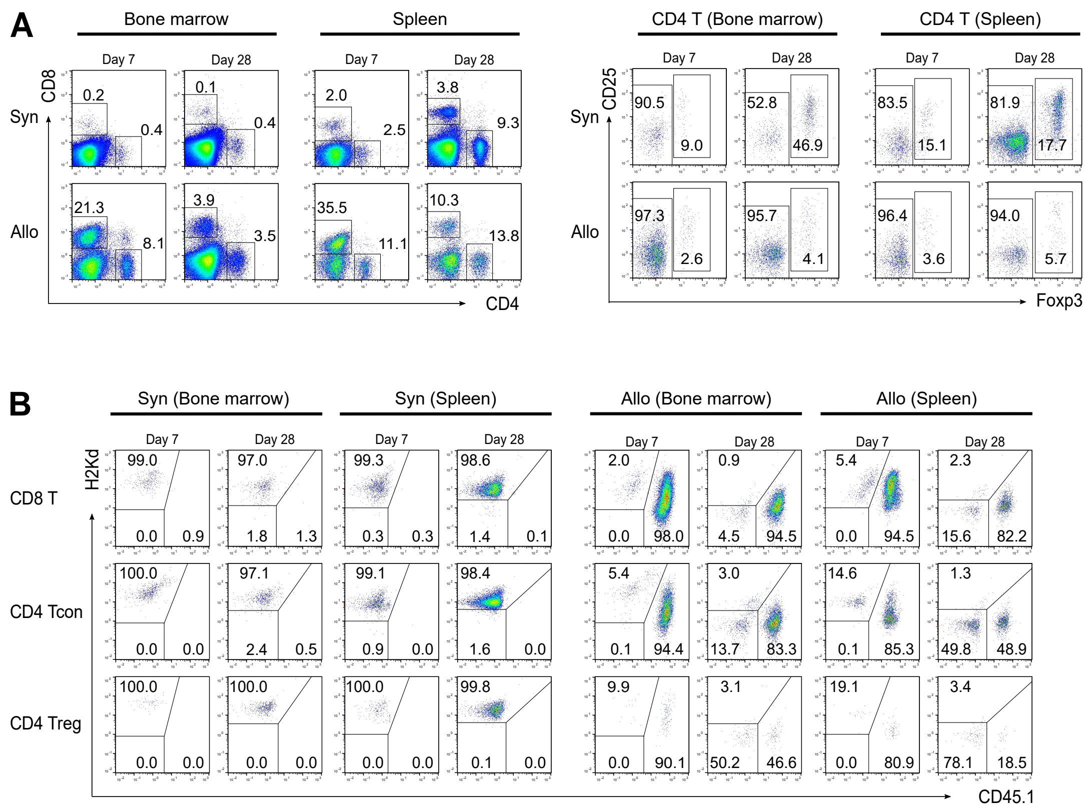

Supplemental Figure 1. Gating strategy used to identify the T-cell origin: host-, graft-, and HSC-derived.

(**A**) Representative flow cytometry plots identifying $CD4^+$ and $CD8^+$ T cell subsets in the bone marrow and spleen of the syngeneic and allogeneic groups. (**B**) Representative flow cytometry plots of T-cell subsets in the bone marrow and spleen of the syngeneic and allogeneic groups for chimerism analysis. $CD8^+$ T-cells, $CD4^+$ Tcons, and $CD4^+$ Tregs in the allogeneic group were separated into host- ($H2Kd^+CD45.1^-$), graft- ($H2Kd^-CD45.1^+$), and HSC- ($H2Kd^-CD45.1^-$) derived cells. HSC, hematopoietic stem cell; Tcon, conventional T-cell; Syn, syngeneic; Allo, allogeneic.

Supplemental Figure 2

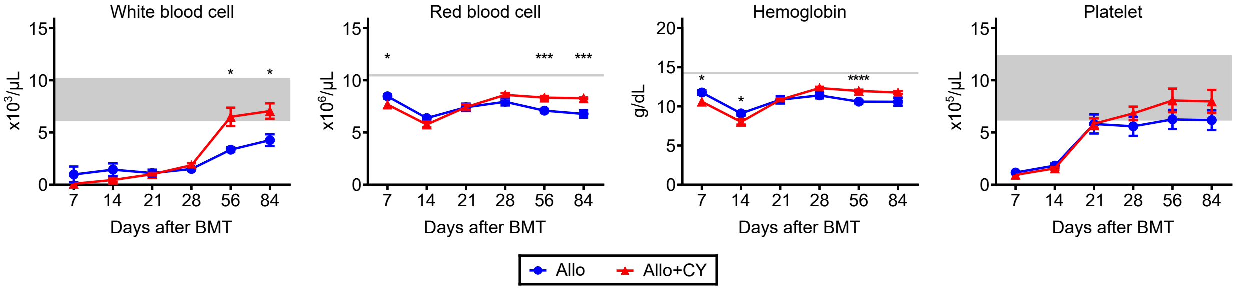

Supplemental Figure 2. PTCy was associated with early recovery peripheral blood cell counts after allogeneic BMT.

Lethally irradiated (10 Gy) BDF1 recipients (H2K$^{b/d}$CD45.2^{+}) received transplants of 5×10^6 Ly 5.1 B6 (H2K$^{b/b}$CD45.1^{+}) splenocytes and 5×10^6 B6 (H2K$^{b/b}$CD45.2^{+}) TCD-BM cells. All recipient mice were injected intraperitoneally with 50 mg/kg cyclophosphamide or vehicle on day 3 after allogeneic BMT. The animals were euthanized on days 7, 14, 21, 28, 56, and 84 after allogeneic BMT to harvest peripheral blood. The kinetics of white blood cells, red blood cells, hemoglobin, and platelet recovery in the peripheral blood after allogeneic BMT (vehicle-treated, n = 48 and PTCy-treated, n = 39). The data were obtained from three independent experiments. Gray bars indicate the mean reference values ± SEM of normal controls (NC, n = 3). *P*-values were determined using the Mann–Whitney *U*-test. $^{*}P < 0.05$, $^{***}P < 0.001$. PTCy, posttransplant cyclophosphamide; CY, cyclophosphamide; BMT, bone marrow transplantation; BDF1, B6D2F1; B6, C57BL/6J; Ly 5.1 B6, CD45.1 C57BL/6J; TCD-BM, T-cell-depleted bone marrow.

Supplemental Figure 3

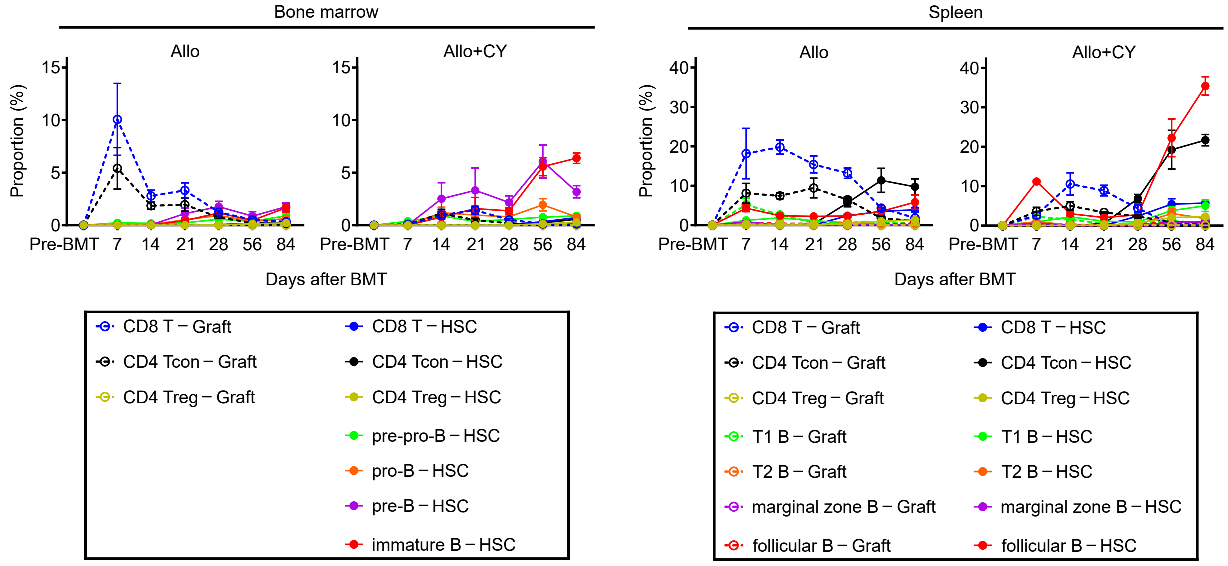

Supplemental Figure 3. Proportions of T and B cells over time after PTCy treatment. Lethally irradiated (10 Gy) BDF1 recipients (H2K$^{b/d}$CD45.2^{+}) received transplants of 5 × 10^{6} Ly 5.1 B6 (H2K$^{b/b}$CD45.1^{+}) splenocytes and 5 × 10^{6} B6 (H2K$^{b/b}$CD45.2^{+}) TCD-BM cells. All recipient mice were injected intraperitoneally with 50 mg/kg cyclophosphamide or vehicle on day 3 post allogeneic BMT (vehicle-treated, n = 30 and PTCy-treated, n = 32). Proportions of graft-derived or HSC-derived T- and B-cell subsets in the bone marrow nucleated cells and splenic lymphocytes of vehicle- and PTCy-treated groups. Graft- and HSC-derived cells were defined as H2Kd^{-}CD45.1^{+} and H2Kd^{-}CD45.1^{-} gated cells, respectively, using flow cytometry. Data from two independent experiments were combined and expressed as the mean ± SEM.

Supplemental Figure 4

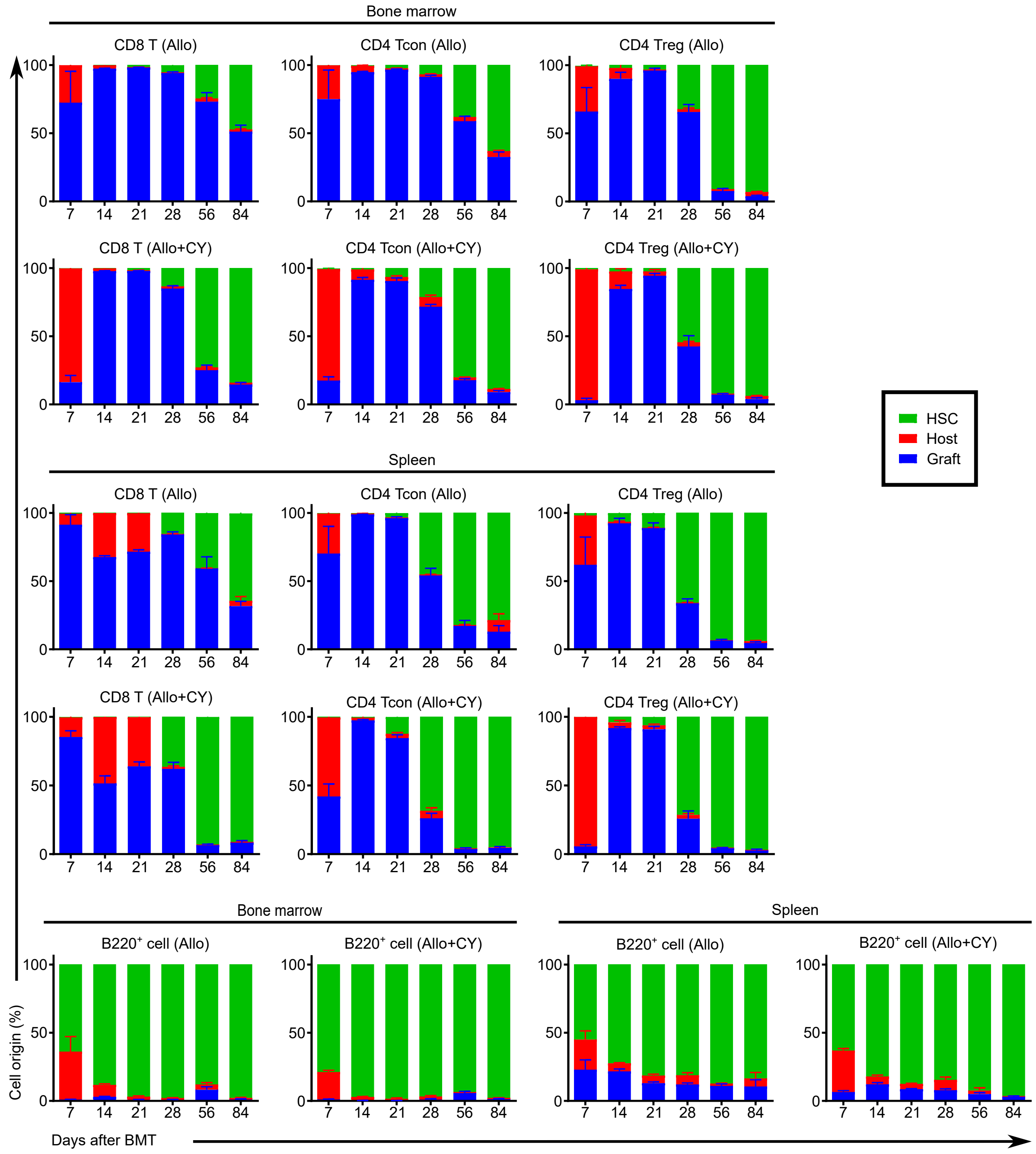

Supplemental Figure 4. Chimerism of T and B cells over time following PTCy treatment. Lethally irradiated (10 Gy) BDF1 recipients ($H2K^{b/d}CD45.2^{+}$) received transplants of 5 × 10^6 Ly 5.1 B6 ($H2K^{b/b}CD45.1^{+}$) splenocytes and 5 × 10^6 B6 ($H2K^{b/b}CD45.2^{+}$) TCD-BM cells (allogeneic group). All recipient mice were injected intraperitoneally with 50 mg/kg cyclophosphamide or vehicle on day 3 after allogeneic BMT (vehicle-treated, n = 30 and PTCy-treated, n = 32). Chimerism analysis of $CD8^{+}$ T cell, $CD4^{+}$ Tcon, $CD4^{+}$ Treg, and $B220^{+}$ cell in the bone marrow and spleen of vehicle- and PTCy-treated groups. Data from two independent experiments were combined and expressed as the mean ± SEM.

Supplemental Figure 5

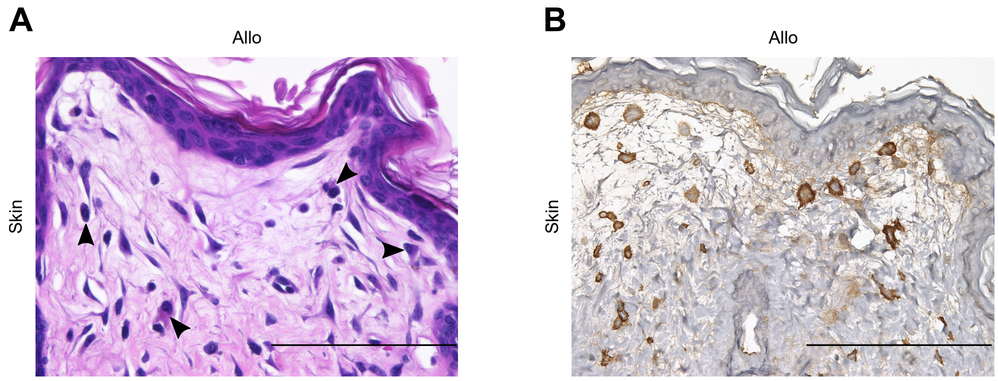

Supplemental Figure 5. IgG-positive plasma cells infiltrated into dermis and subcutaneous fat layer in allogeneic vehicle-treated recipients.

Lethally irradiated (10 Gy) BDF1 recipients ($H2K^{b/d}CD45.2^{+}$) received transplants of 5 × 10^6 splenocytes and 5 × 10^6 TCD-BM cells from B6 mice ($H2K^{b/b}CD45.2^{+}$). All recipient mice were injected intraperitoneally with 50 mg/kg cyclophosphamide or vehicle on day 3 after allogenic BMT. The recipient mice were euthanized and the skin was harvested 12 weeks posttransplant. (**A**) A representative image of the skin from allogeneic vehicle-treated recipients stained with hematoxylin and eosin (scale bar = 100 µm, original magnification ×600). Arrows indicate plasma cells with abundant cytoplasm and eccentric nuclei. (**B**) A representative image of skin from allogeneic vehicle-treated recipients with IgG immunostaining (scale bar = 100 µm, original magnification ×600).

Supplemental Figure 6

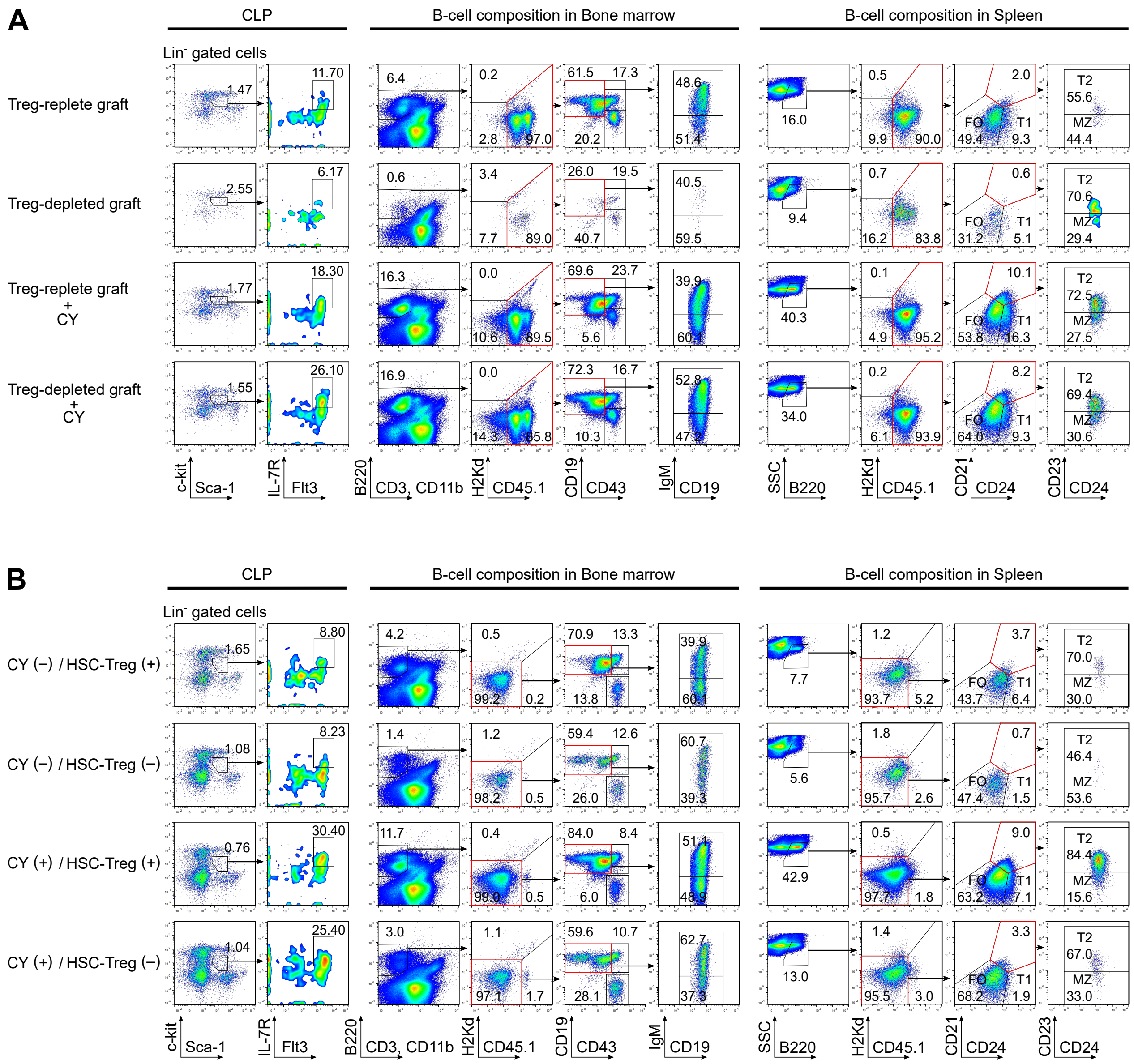

Supplemental Figure 6. Depletion of HSC-derived Tregs, not graft-derived Tregs, was associated with impaired B-cell development after PTCy-treated BMT.

(**A** and **B**) Representative flow cytometry plots of CLP, B220^{+} cell subsets, and chimerism in the bone marrow and spleen on day 56 after allogeneic BMT in the experiment in which graft-derived Tregs were depleted (**A**) and in the experiment in which HSC-derived Tregs were depleted (**B**). CLP, common lymphoid progenitor; Lin, lineage.
